# Retrieval analysis of PEEK rods pedicle screw system: three cases analysis

**DOI:** 10.1186/s12891-024-07600-0

**Published:** 2024-06-22

**Authors:** Xiaoduo Xu, Lei Wang, Jingming Wang, Xiuchun Yu, Weimin Huang

**Affiliations:** Orthopedic Department, 960 Hospital of People’s Liberation Army, NO.25 Shifan Road, Jinan, Shandong 250031 China

**Keywords:** PEEK rods, Revision surgery, Histological response, Fatigue testing, Retrieval analysis

## Abstract

**Purpose:**

To analyze the characteristics of PEEK rods retrieved in vivo, specifically their wear and deformation, biodegradability, histocompatibility, and mechanical properties.

**Method:**

Six PEEK rods were retrieved from revision surgeries along with periprosthetic tissue. The retrieved PEEK rods were evaluated for surface damage and internal changes using Micro-CT, while light and electron microscopy were utilized to determine any histological changes in periprosthetic tissues. Patient history was gathered from medical records. Two intact and retrieved PEEK rods were used for fatigue testing analysis by sinusoidal load to the spinal construct.

**Results:**

All implants showed evidence of plastic deformation around the screw-rod interface, while the inner structure of PEEK rods appeared unchanged with no visible voids or cracks. Examining images captured through light and electron microscopy indicated that phagocytosis of macrophages around PEEK rods was less severe in comparison to the screw-rod interface. The results of an energy spectrum analysis suggested that the distribution of tissue elements around PEEK rods did not differ significantly from normal tissue. During fatigue testing, it was found that the retrieved PEEK rods cracked after 1.36 million tests, whereas the intact PEEK rods completed 5 million fatigue tests without any failure.

**Conclusion:**

PEEK rods demonstrate satisfactory biocompatibility, corrosion resistance, chemical stability, and mechanical properties. Nevertheless, it is observed that the indentation at the junction between the nut and the rod exhibits relatively weak strength, making it susceptible to breakage. As a precautionary measure, it is recommended to secure the nut with a counter wrench, applying the preset torque to prevent overtightening.

**Supplementary Information:**

The online version contains supplementary material available at 10.1186/s12891-024-07600-0.

## Introduction

Lumbar degenerative diseases are the most common spinal condition, and it has been reported that a total of 266 million individuals (3.63%) worldwide were found to have lumbar degenerative diseases annually [1]. Lumbar fusion procedure aided by titanium rod is a popular protocol with relatively good evidence of effectiveness [2]. However, the increased application of this technique has led to the emergence of some related complications, including fusion failure, screw loosening, titanium rod breakage, and adjacent segment degeneration [3, 4].

In recent years, polyetheretherketone (PEEK) materials have gained clinical significance due to their exceptional biocompatibility, corrosion resistance, and stability [5–7]. PEEK rods system is approved by the FDA for posterior lumbar spine surgery as a semi-rigid internal fixation system since 2007. Compared to the traditional titanium rod, the elastic modulus of PEEK rod is lower, which is similar to that of cortical bone with an elastic modulus of 3.2GPa [8, 9]. The use of PEEK rods enabled an increase in stress on the anterior column while simultaneously reducing stress on the posterior column. This technique is known to encourage intervertebral bony fusion and alleviate pressure in the bone-screw interface, ultimately resulting in improved outcomes and less postoperative complications [9–12]. Furthermore, PEEK rods are radiolucent and do not create artifacts in MRI and CT examination, which is helpful to determine the postoperative evaluation [13, 14].

Over time, the use of PEEK rods in clinical practice has grown, leading to a rise in relevant reports [15–22]. However, there is a lack of research concerning complications and retrieval analysis. One previous study conducted a retrieval analysis of PEEK rods, revealing the most common issue to be permanent indentations caused by the nuts and screws [23]. However, this study lacks the use of electron microscopy for analyzing periprosthetic tissue, as well as fatigue testing for retrieving PEEK rods. The need for additional information on the performance of PEEK rods in vivo is significant. This study aims to conduct a comprehensive analysis using Micro-CT, fatigue testing, and both optical and electron microscopy observation to enhance our understanding of the biodegradability, histocompatibility, and mechanical properties of PEEK rods.

## Materials and methods

### Retrieved PEEK rods systems

The retrieval analysis involved six PEEK rods and periprosthetic tissue samples taken from three patients. The revision surgeries were performed at the local hospital between June 2015 and March 2017. The patients voluntarily provided consent to donate their implanted PEEK rods for retrieval analysis. It should be noted that the revision surgeries were conducted as routine procedures and were not part of a prospective clinical trial.

The retrieved devices used in revision surgery included six PEEK rods, all of which were of the same design (Wego, China). These PEEK rods were pre-curved in the sagittal plane to account for lumbar lordosis. The length of each retrieved PEEK rod varied based on the index level.

The transpedicular screws were composed of a medical-grade titanium alloy (Ti-6Al-4 V). All the pedicle screws had identical configurations, with a diameter of 6.5 mm and a screw length of 45 mm. The relevant information of the internal fixation system was presented in Table 1.

**Table 1:** The type and dimensions of the PEEK rods system

Sample	Specification(mm)	Type
PEEK rods	6.35** × **7.2** × **100	GB2Z
Polyaxial pedicle screw	4.5** × **35	GB2Z
Top screw	6.35	GB2Z

The current retrieval analysis included two females and one male, with two cases diagnosed with lumbar spinal stenosis and one case with lumbar disc herniation. The clinical details for the patients in this study were presented in Table 2.

**Table 2:** Clinical characteristics of the enrolled cases

	Case 1	Case 2	Case 3
Age (years)	61	49	56
Gender	Female	Male	Female
Diagnosis	Lumbar spinal stenosis	Lumbar disc herniation	Lumbar spinal stenosis
Index levels	L3-5	L2-5	L2-4
In vivo duration	7 months	16 months	37 months
Chief complaint before revision surgery	Back pain	Back and leg pain	Back and leg pain
Reasons for revision surgery	Cage subsidence	Cage subsidence	Screws loosening, bilateral PEEK rods fracture

### Surface damage and internal structure change assessment

Micro-CT (Inveon, Siemens, Germany) was employed to examine the surface damage of the retrieved PEEK rods. This involved inspecting wear, fatigue cracks, plastic deformation, and delamination. To gain a deeper understanding of the entire structure of the PEEK rods, the rods were divided into five segments based on average length. We then conducted three-dimensional reconstructions using Micro-CT to observe any internal alterations present within the PEEK rods. Quantitative analysis was performed on the indentation density. Two intact PEEK rods were examined under the same conditions for comparison. The evaluation of all retrieved PEEK rods was conducted by the same investigator.

### Histologic analysis of periprosthetic tissues

During the revision surgeries, two pieces of tissue were extracted from the periprosthetic tissue encompassing the PEEK rods and screw-rod interface. These tissues measured approximately 5 × 5 mm each. To investigate the tissues more thoroughly, each piece was divided into two parts. One part was examined using an optical microscope, while the other was studied with an electron microscope.

#### Preparation of sample for optical microscopy

After obtaining the sample, it was rinsed with chilled PBS. The tissues were then cut into 10 mm×10 mm×5 mm pieces and fixed in 10% formalin for 24 h. Following cleaning, dehydration, clearing, and paraffin impregnation, two representative tissues were embedded in paraffin blocks and marked for identification. The tissue was then sectioned into sections measuring 6–10 μm using a microtome. Serial 6 μm sections were procured, de-waxed, and stained with 0.2% ammonia water in combination with hematoxylin and eosin (H&E).

Tissue sections were imaged under using a transmitted light microscope (Axio Scope A1, CarlZeissAG, Germany).

#### Preparation of samples for electron microscopy

The retrieved tissue’s surface was rinsed with a phosphate buffer. Specimens were then fixed in a 2.5 ~ 5% glutaraldehyde solution for one hour at 4℃. The unbound glutaraldehyde was washed off by rinsing the specimens three times. Next, the specimens were fixed using a 1% osmic anhydride and buffer solution (0.2 mol/L phosphate). The prepared specimens were then dehydrated, underwent dry and vacuum coating processing, and finally observed using the electron microscope (JSM-6610LV, JEOL, Japan).

Ion distribution was analyzed by energy spectrum analysis using an electron microscope.

### Fatigue testing of implanted and unused PEEK rods

**Fig. 1:**
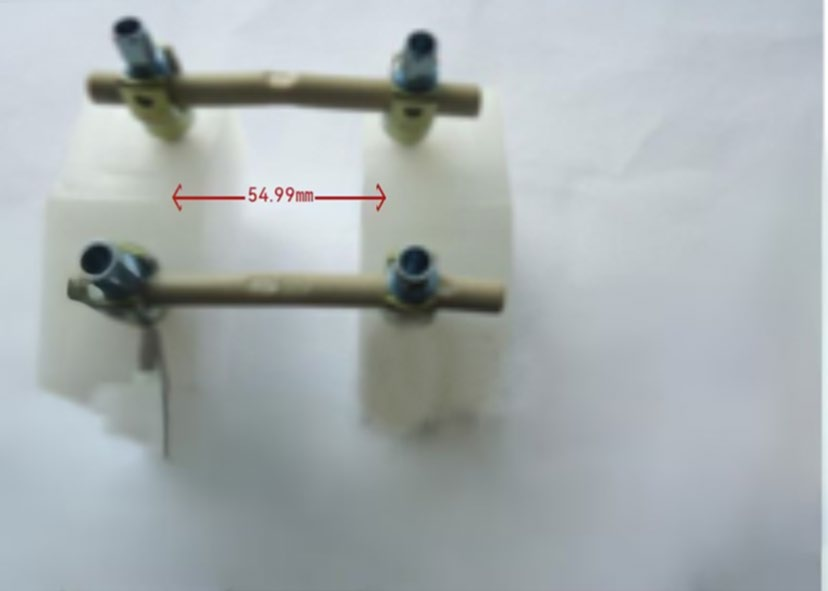
Stiffness measurement of the retrieval PEEK rods prior to fatigue testing (Locked at the original indentation)

**Fig. 2:**
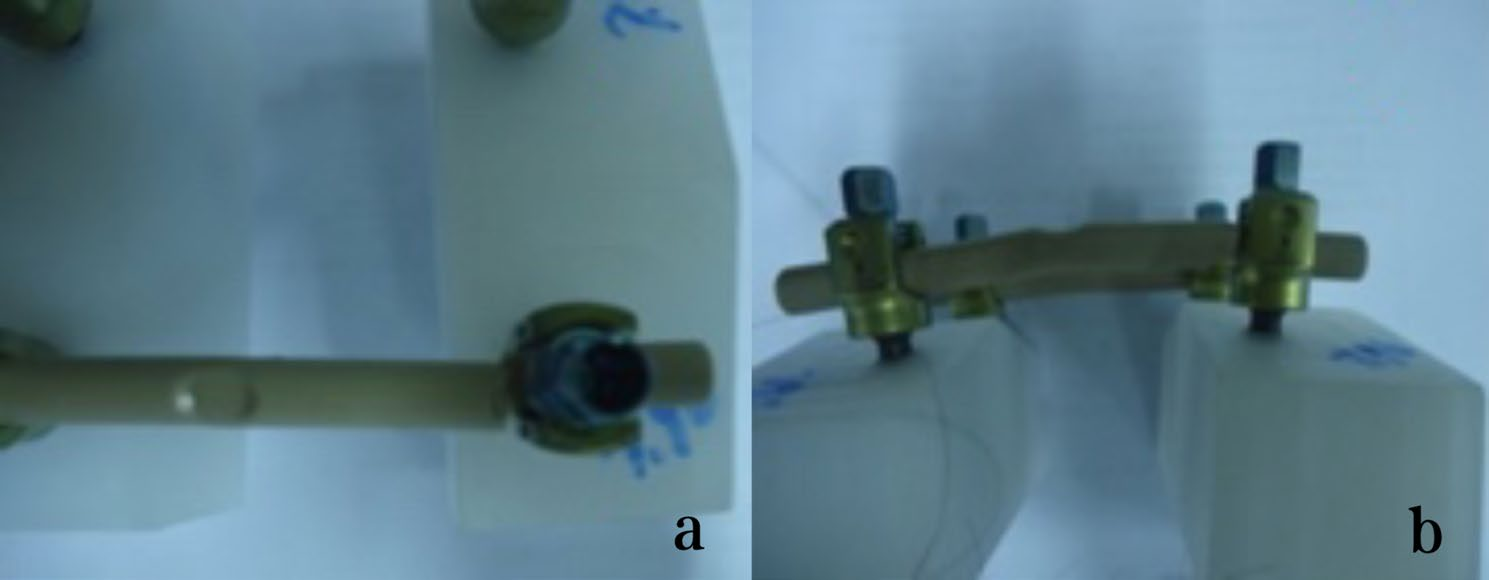
PEEK rods fracture at the screw-rod interface when adjusting stiffness by static load

**Fig. 3:**
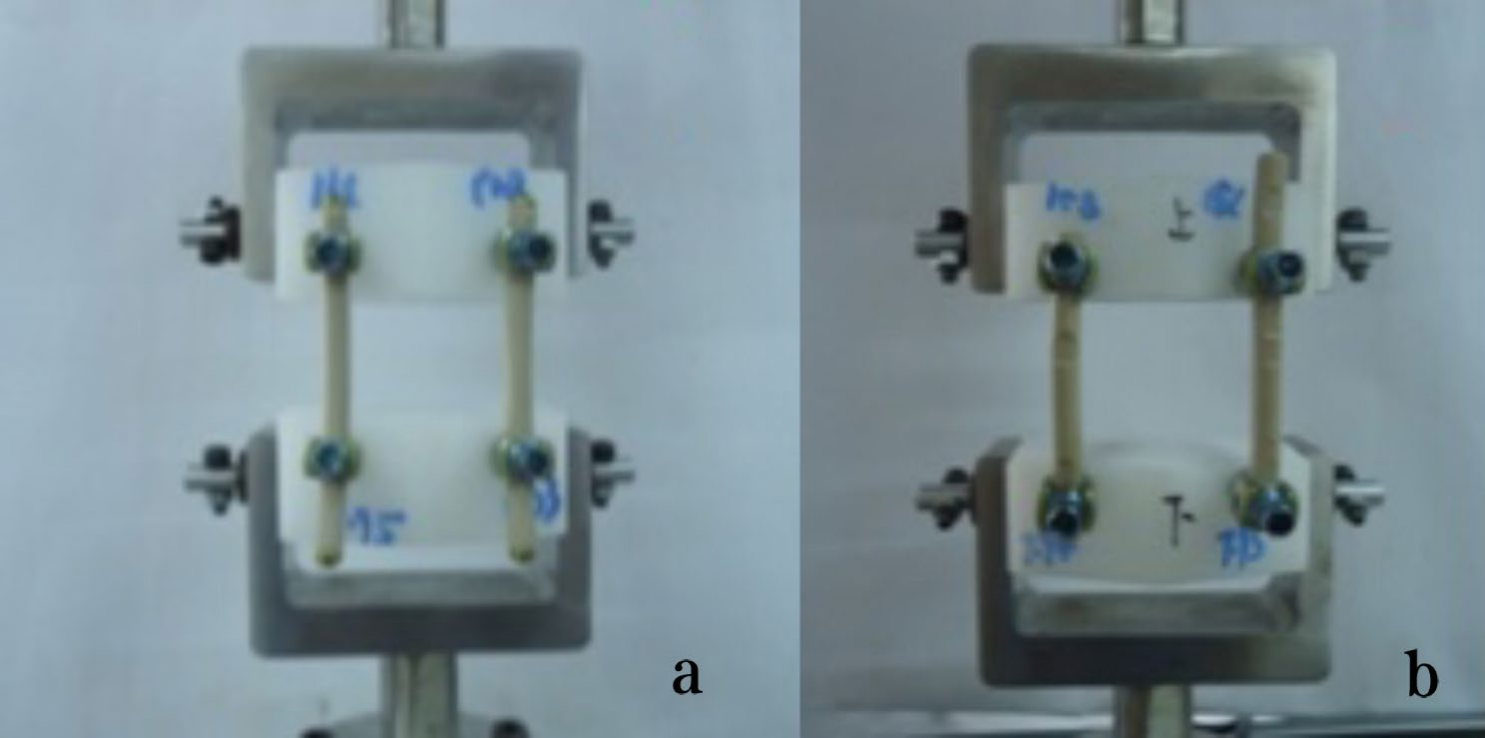
A lumbar bilateral UHMWPE Block (**a**: Unused PEEK rods, **b**: Implanted PEEK rods)

The fatigue test was conducted at room temperature using two retrieval PEEK rods and two intact PEEK rods, following the ASTM F1717-13 Standard Test Methods for Spinal Implant Constructs in a Vertebrectomy Model [24]. According to the ASTM F 1717 standard, the testing of spinal implant assemblies involves two Ultra High Molecular Weight Polyethylene (UHMWPE) test blocks to simulate vertebral model with a significant gap between them to simulate intervertebral space. The UHMWPE material used for the test blocks have a tensile breaking strength of 40 ± 3 MPa. By using UHMWPE test blocks, the variability in bone properties and morphometry can be eliminated. The test was conducted using the Instron E10000 test machine (Instron UK Ltd. High Wycombe, United Kingdom) and the Wave Matrix software (Version 2.1). The PEEK rods and pedicle screws used in the fatigue test were of the same design (Wego, China). The specifications of the PEEK rods were 6.35 × 7.2 × 100 mm, and the specifications of the pedicle screws were 6.5 × 45 mm. To assemble, we locked the fastening screws at the original indentation of the implanted PEEK rods as shown in Fig. 1, with an assembled pitch of 54.99 mm. The stiffness of the rods was adjusted by applying a static load. During this adjustment process, it was discovered that one of the implanted PEEK rods had a crack, as shown in Fig. 2. To ensure accurate results, the assembled pitch was adjusted to a measurement of 51.21 mm. The PEEK rod was then locked in a position that avoided the original indentation, as depicted in Fig. 3. The fatigue testing procedure involved subjecting the spinal construct to a sinusoidal load with a load ratio of 0.1, a maximum load of 115 N, and a frequency of 2 Hz. The value of 115 N was determined through the execution of fatigue life curve assessments on intact PEEK rods, representing the load threshold at which 5 million cycles were attained without failure.

## Results

### Implant analysis

**Fig. 4:**
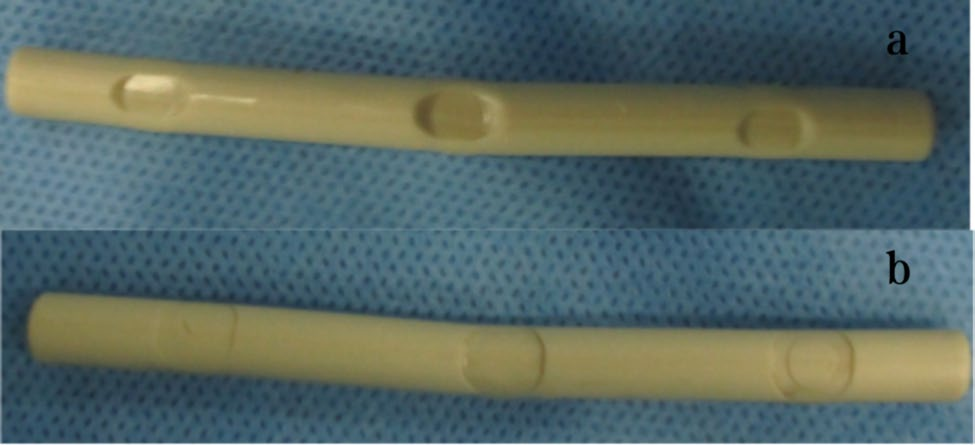
Plastic indentation of retrieval PEEK rod on the nut (**a**) and screw (**b**) interface

During the revision surgery, it was discovered that one case had experienced bilateral PEEK rod fractures. Additionally, plastic indentation was observed at the screw-rod interface for all six PEEK rods, as shown in Fig. 4. Despite this, no visible scratches were detected on the implanted PEEK rods.

**Fig. 5:**
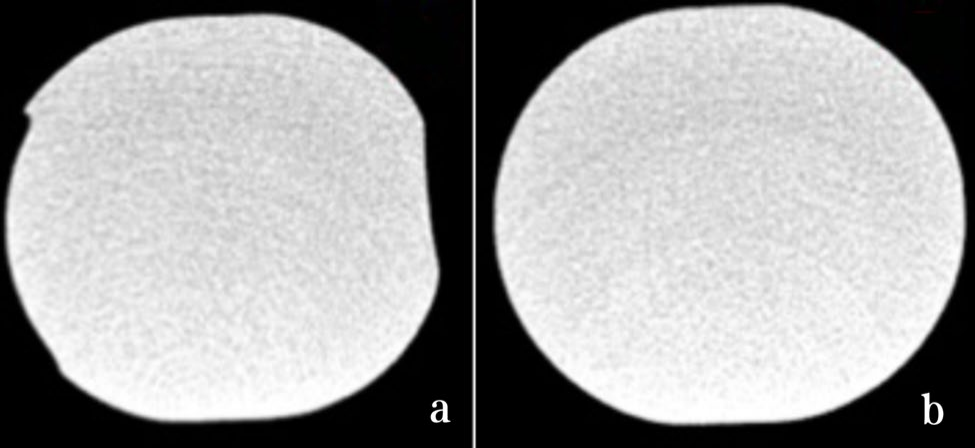
Micro-CT scans the retrieval PEEK rod at the indentation (**a**) and the intact PEEK rod (**b**)

**Fig. 6:**
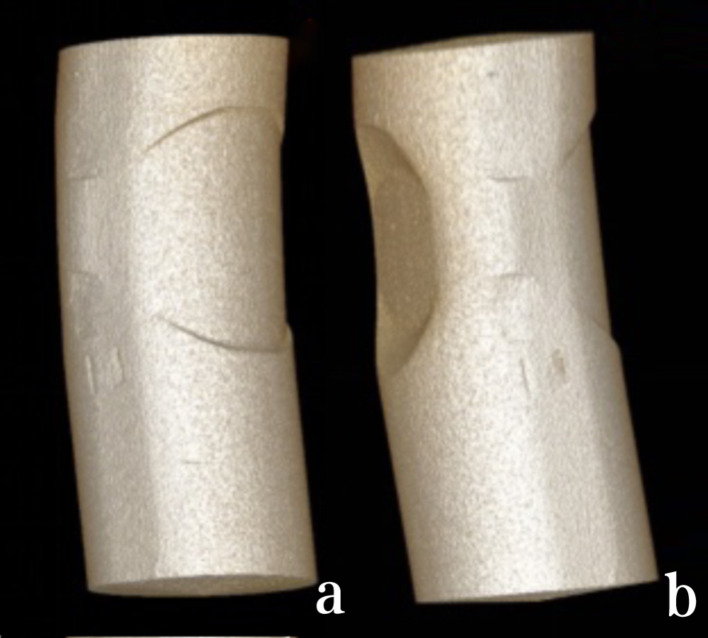
Micro-CT scans and 3D reconstruction of retrieved PEEK rods

**Fig. 7:**
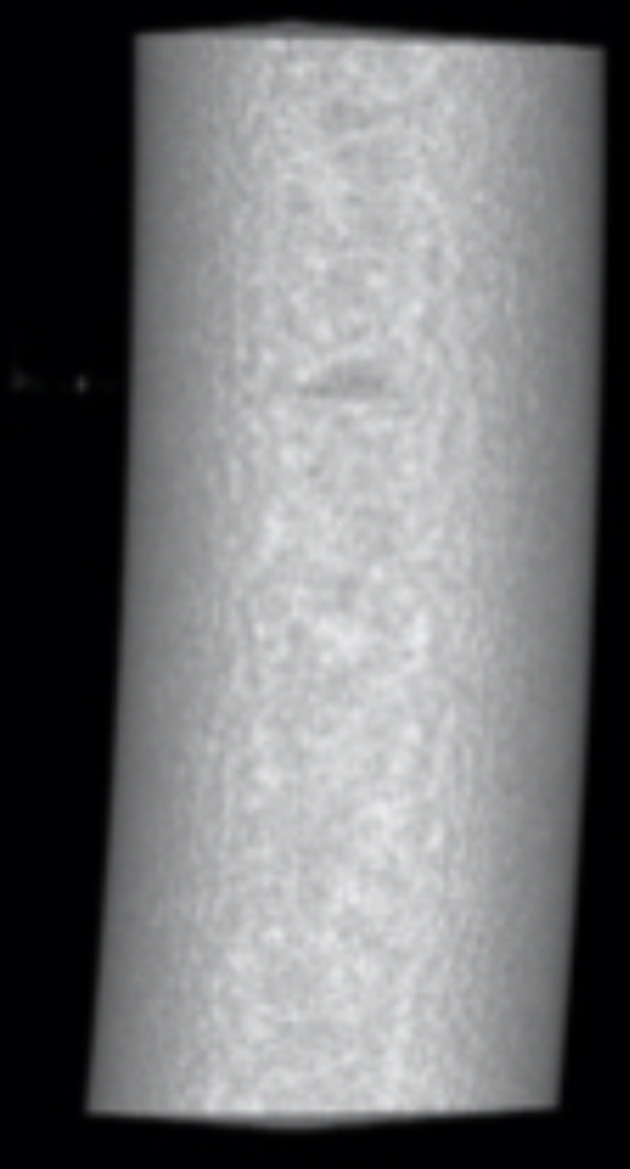
Micro-CT scans and 3D reconstruction of intact PEEK rods

Upon conducting micro-CT examination, it was determined that the implanted PEEK rods showed no signs of wear, fatigue cracks, pitting, corrosion or embedded debris. However, there was evidence of plastic deformation at the screw-rod interface. Nevertheless, the internal structure of the implanted PEEK rods remained unchanged when compared to intact PEEK rods. Furthermore, quantitative analysis of density at the indentation interface revealed no discernable difference compared to the intact PEEK rods (Figs. 5, 6 and 7).

### Tissue analysis

**Fig. 8:**
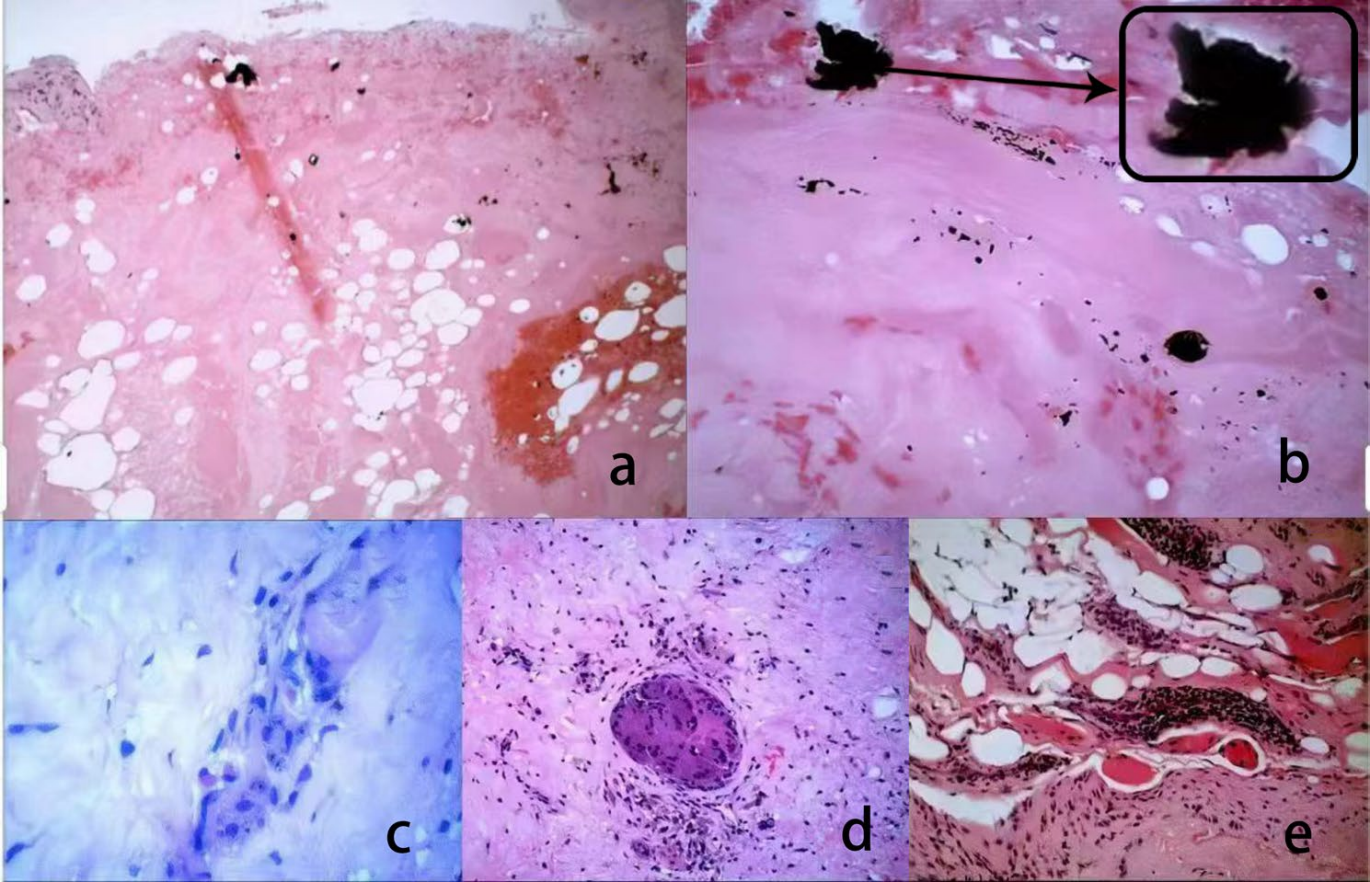
Retrieved tissue analyzed by optical microscope **a**: Periprosthetic tissue around PEEK rods **b**: Metallic foreign bodies were found in periprosthetic tissue surrounding the screw-rod interface **c**: Representative image of macrophage phagocytosis in periprosthetic tissue surrounding PEEK rods **d**: Representative image of macrophage phagocytosis in periprosthetic tissue surrounding the screw-rod interface **e**: An inflammatory reaction in periprosthetic tissue around the screw-rod interface

The retrieval analysis of tissues around PEEK rods and screw-rod interfaces reveals several noteworthy observations. Using optical microscopy, signs of bleeding, fibrinous exudation, macrophage phagocytosis, and metallic foreign bodies were detected. It was observed that the tissue around the screw-rod interface had more intense macrophage phagocytosis, and a greater prevalence of metallic foreign bodies, when compared to the tissue around PEEK rods. Moreover, vascular inflammation was limited to the regions surrounding the screw-rod interface. A visual representation of these findings was showed in Fig. 8.

**Fig. 9:**
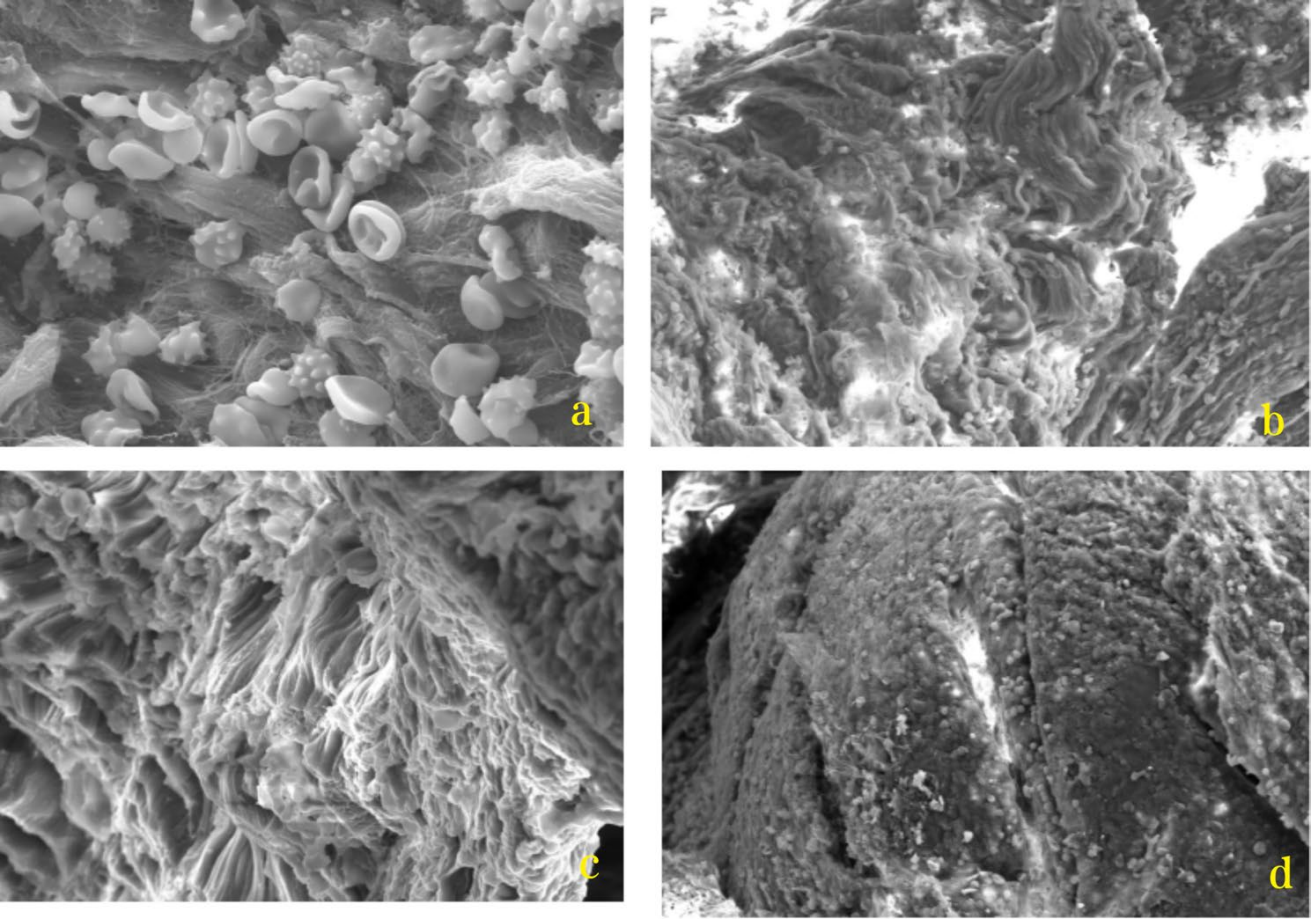
Periprosthetic tissue around PEEK rods observed by electron microscopy. **a**: Normal red blood cells **b**: Granulation tissues **c**: Fibrous tissues **d**: Interstitial tissue hyperemia

**Fig. 10:**
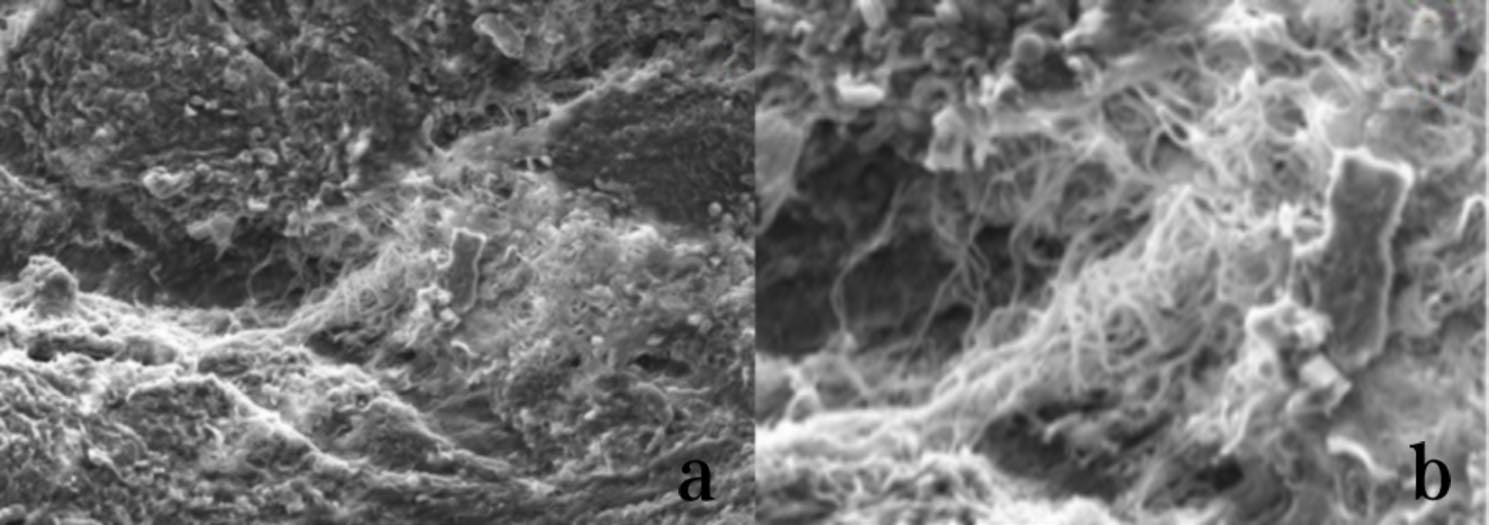
Periprosthetic tissue around the screw-rod interface observed by electron microscopy. **a**: Irregular sheet material **b**: Filamentous material

Electron microscopy revealed that none of the three observed patients had any foreign bodies or fragments in their periprosthetic tissues. The tissue analysis showed normal red blood cell count and hyperemia in interstitial, granulation, and fibrous tissue (Fig. 9). However, irregular sheet material and filamentous substances were present around the screw-rod interface, possibly indicating metal debris from the screw. Despite this finding, no inflammatory reaction was observed in the periprosthetic tissues (Fig. 10).

**Fig. 11:**
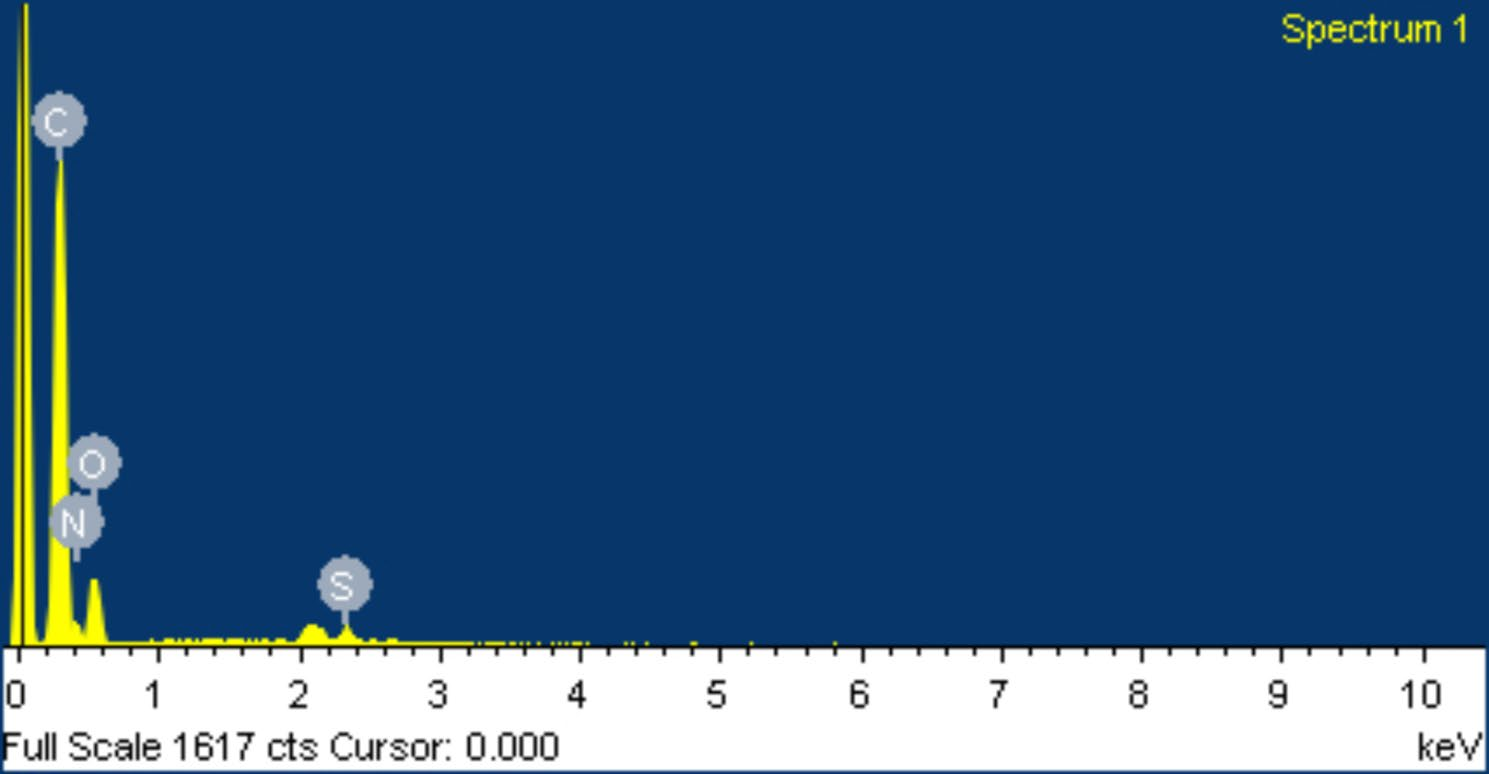
Energy spectrum analysis of periprosthetic tissue surrounding PEEK rods observed by electron microscopy

**Fig. 12:**
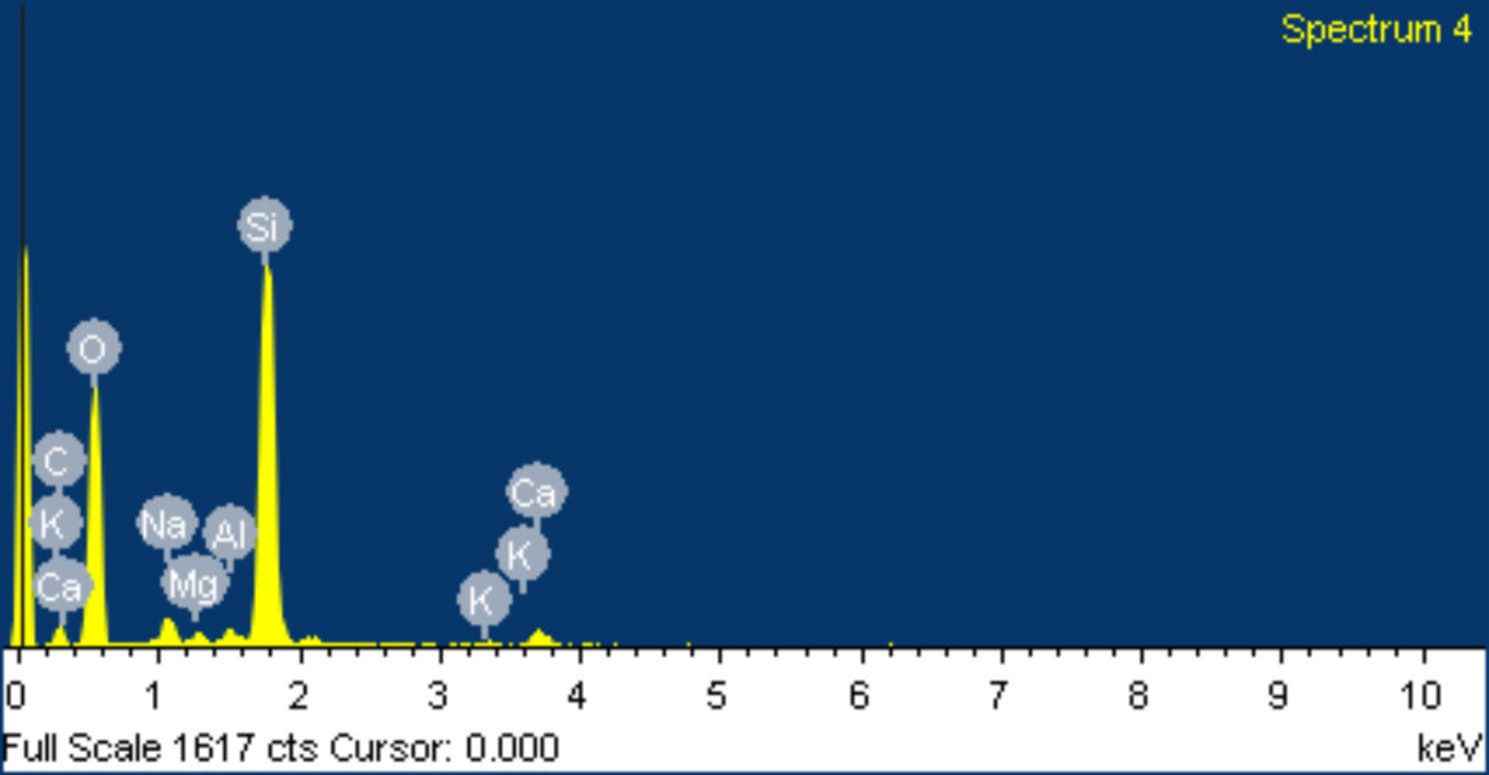
Energy spectrum analysis of periprosthetic tissues surrounding the screw-rod interface observed by electron microscopy

Upon conducting an energy spectrum analysis of tissue surrounding PEEK rods, it was discovered that the primary elements present in the tissue were carbon, oxygen, nitrogen, and sulfur. Carbon was found to have the highest weight and atom percentages. However, it was observed that the ion distribution in the tissue surrounding the screw-rod interface was different from that of typical tissue. This particular area was primarily composed of various metal elements, including silicon, sodium, aluminum, magnesium, and calcium. Of the metal elements, silicon had the highest weight and atom percentages as depicted in Figs. 11 and 12.

### Fatigue testing

**Fig. 13:**
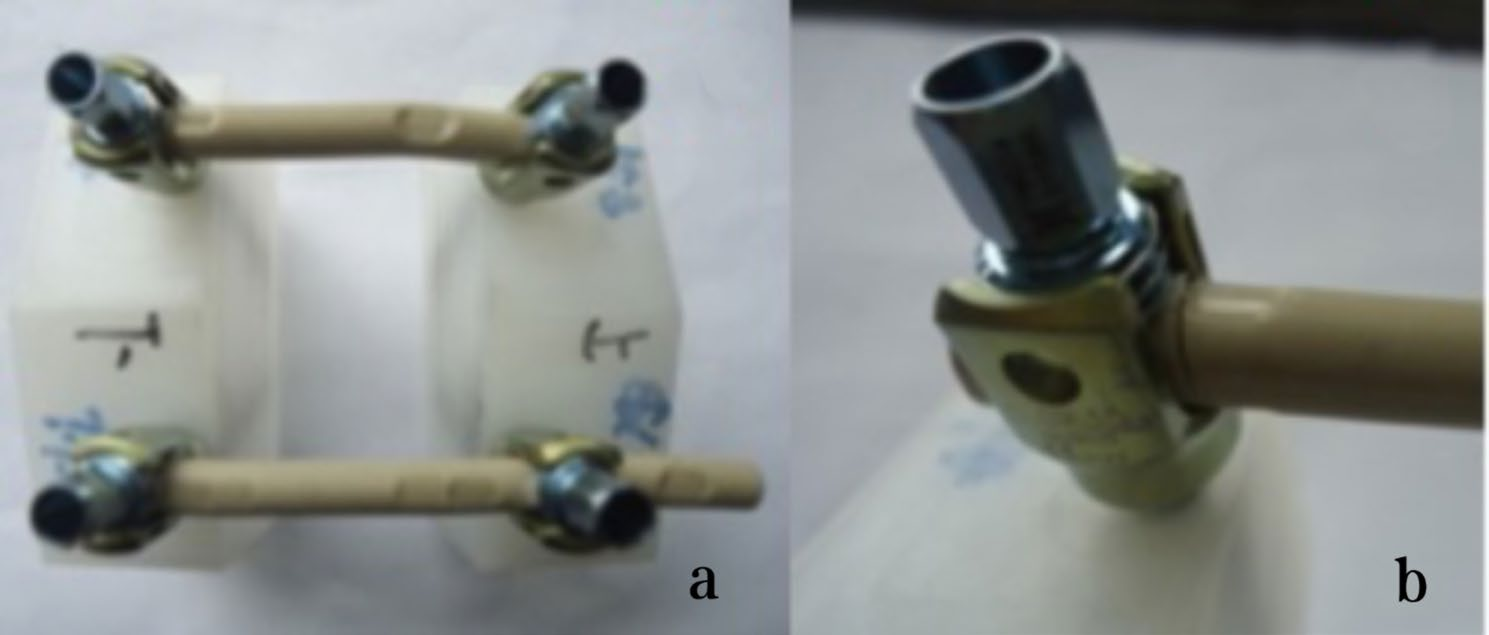
Fracture of the retrieval PEEK rod occurred after 1.36 million cycles of fatigue testing

Table 3 presented the results of the fatigue testing. The intact PEEK rods displayed remarkable durability, remaining intact even after exposure to 5 million cycles of fatigue testing. Conversely, the implanted PEEK rods suffered from fractures at the nut-screw indentation after just 1.36 million cycles of testing (Fig. 13).

**Table 3:** Fatigue testing

Test group	Locking torque (Nm)	Rigidity (*N*/mm)	Cycle index (million)	Results
Retrieval PEEK rods	8	4.649	1.36	Fracture
Intact PEEK rods	8	5.574	5	Intact

## Discussion

This study examines the surface and internal structural changes of PEEK rods, as well as histological responses retrieved from surgeries. Additionally, analysis of the mechanical properties of these rods has been conducted. This research provides an in vivo analysis of the characteristics, mechanical properties, and histological responses of PEEK rods.

The observations made in this study are comparable to the findings reported in prior research on the revision of the Dynesys system. In a study conducted by Neukamp [24], surface damage was observed in five Dynesys revision surgery cases with a mean implanted time of 2.86 years. Each polyurethane (PCU) spacer displayed varying degrees of damage including scratches and plastic deformation, and abrasive sections were discovered in three cases. Additional analysis of surrounding tissue revealed large impurity particles, debris, and phagocytosis of macrophages. Similarly, Shen reported on a retrieval analysis of Dynesys in four patients. Over the course of 9–19 months of implantation, the retrieved components showed good biostability in both PCU spacers and polyethyleneterephthalate (PET) cords upon explanation [25]. The assessment of retrieved implants has yielded vital knowledge regarding device functionality and durability.

This study found clear evidence of indentation (plastic deformation) on the screw-rod interface in all revision cases. However, retrieved rods showed no signs of scratches, wear, fatigue cracks, or pitting. Micro-CT scans further confirmed these findings, showing no internal changes like voids, cracks, embedded chips, or corrosion in the areas of indentation.

Observations using electron and optical microscopy demonstrated superior histocompatibility of PEEK rods. The samples showed signs of normal red blood cell shapes, granulation tissue, fibrous tissue, hyperemia of interstitial tissue, fibrinous exudate, and chronic inflammatory cell infiltration. However, there were more foreign bodies and a greater abundance of macrophage phagocytosis in the tissue surrounding the screw-rod interface compared to the tissue around PEEK rods. The energy spectrum analysis from electron microscopy revealed that there were higher levels of metal ions present in the tissue surrounding the screw-rod interface. The weight and atomic percentages of silicone were found to be at their highest point. On the other hand, tissues surrounding the PEEK rods had ion distribution that was more similar to normal tissues, with carbon and nitrogen occupying the peak of weight and atomic percentage.

The fatigue testing indicated that the retrieval PEEK rods broke at the interface of the nut-screw indentation, aligning with the intraoperative findings of case 3. These findings imply that the indentation may be the weakest point where the PEEK rod is prone to break after installation. Previous biomechanical assessments have demonstrated that the PEEK rod system is capable of providing adequate grip strength without experiencing deformation at a designated torque [11]. However, it is important to note that the designated torque requires the use of a counter wrench. In the absence of a counter wrench during surgery, there is the potential for increased torque and deeper indentation, which can ultimately result in an increased likelihood of PEEK rod fractures.

Some limitations should be noted when interpretating the results in the current study. Firstly, similar to other retrieval studies, this work was limited to a small number of cases. Secondly, the observation of the tissue using both light microscope and electron microscope only captured a portion of the tissue surrounding the PEEK rod. Therefore, it could not be conclusively determined if this selected tissue was the most representative, which could potentially impact the accuracy of the observation results. Thirdly, although the current medical PEEK materials are primarily supplied by manufacturer Invibio (from UK), different manufacturing processes of different companies may lead to product inconsistencies when it is made into PEEK rods. Additionally, the in vivo performance of rods with varying diameters may be also different. Therefore, it is important to acknowledge that the PEEK rods in this study may not represent all PEEK rod products.

## Conclusion

PEEK rods demonstrate satisfactory biocompatibility, corrosion resistance, chemical stability, and mechanical properties. Nevertheless, it is observed that the indentation at the junction between the nut and the rod exhibits relatively weak strength, making it susceptible to breakage. As a precautionary measure, it is recommended to secure the nut with a counter wrench, applying the preset torque to prevent overtightening.

### Electronic supplementary material

Below is the link to the electronic supplementary material.

Supplementary Material 1

## Data Availability

The data sets used and/or analyzed during the current study are available from the corresponding author on reasonable request.

## References

[1] Ravindra VM, Senglaub SS, Rattani A, Dewan MC, Härtl R, Bisson E, Park KB, Shrime MG (2018). Degenerative lumbar spine disease: estimating Global Incidence and Worldwide volume. Glob Spine J.

[2] Martin BI, Mirza SK, Spina N, Spiker WR, Lawrence B, Brodke DS (2019). Trends in lumbar Fusion Procedure Rates and Associated Hospital costs for degenerative spinal diseases in the United States, 2004 to 2015. Spine.

[3] Kalakoti P, Missios S, Maiti T, Konar S, Bir S, Bollam P, Nanda A (2016). Inpatient outcomes and Postoperative complications after primary versus revision lumbar spinal Fusion surgeries for degenerative lumbar disc disease: a National (Nationwide) Inpatient Sample Analysis, 2002–2011. World Neurosurg.

[4] Chotai S, Parker SL, Sivaganesan A, Sielatycki JA, Asher AL, McGirt MJ, Devin CJ (2015). Effect of complications within 90 days on patient-reported outcomes 3 months and 12 months following elective surgery for lumbar degenerative disease. Neurosurg Focus.

[5] Panayotov IV, Orti V, Cuisinier F, Yachouh J (2016). Polyetheretherketone (PEEK) for medical applications. J Mater Sci Mater Med.

[6] Skirbutis G, Dzingutė A, Masiliūnaitė V, Šulcaitė G, Žilinskas J (2018). PEEK polymer’s properties and its use in prosthodontics. A review. Stomatologija.

[7] Muthiah N, Yolcu YU, Alan N, Agarwal N, Hamilton DK, Ozpinar A (2022). Evolution of polyetheretherketone (PEEK) and titanium interbody devices for spinal procedures: a comprehensive review of the literature. Eur Spine J.

[8] Ahn YH, Chen WM, Lee KY, Park KW, Lee SJ (2008). Comparison of the load-sharing characteristics between pedicle-based dynamic and rigid rod devices. Biomed Mater.

[9] Ponnappan RK, Serhan H, Zarda B, Patel R, Albert T, Vaccaro AR (2009). Biomechanical evaluation and comparison of polyetheretherketone rod system to traditional titanium rod fixation. Spine J.

[10] Turner JL, Paller DJ, Murrell CB (2010). The mechanical effect of commercially pure titanium and polyetheretherketone rods on spinal implants at the operative and adjacent levels. Spine (Phila Pa 1976).

[11] Gornet MF, Chan FW, Coleman JC, Murrell B, Nockels RP, Taylor BA, Lanman TH, Ochoa JA (2011). Biomechanical assessment of a PEEK rod system for semi-rigid fixation of lumbar fusion constructs. J Biomech Eng.

[12] Selim A, Mercer S, Tang F (2018). Polyetheretherketone (PEEK) rods for lumbar Fusion: a systematic review and Meta-analysis. Int J Spine Surg.

[13] Di Maggio B, Sessa P, Mantelli P, Maniscalco P, Rivera F, Calori GM, Bisogno L, Scaravilli G, Caforio M (2017). PEEK radiolucent plate for distal radius fractures: multicentre clinical results at 12 months follow up. Injury.

[14] Warburton A, Girdler SJ, Mikhail CM, Ahn A, Cho SK (2020). Biomaterials in spinal implants: a review. Neurospine.

[15] Zhao Y, Xu B, Qi L, Li C, Yue L, Yu Z, Wang S, Sun H (2022). Hybrid surgery with PEEK rods for lumbar degenerative diseases: a 2-year follow-up study. BMC Musculoskelet Disord.

[16] Ormond DR, Albert L, Das K (2016). Polyetheretherketone (PEEK) rods in lumbar spine degenerative disease: a Case Series. Clin Spine Surg.

[17] De Iure F, Bosco G, Cappuccio M, Paderni S, Amendola L (2012). Posterior lumbar fusion by peek rods in degenerative spine: preliminary report on 30 cases. Eur Spine J.

[18] Huang W, Chang Z, Song R, Zhou K, Yu X (2016). Non-fusion procedure using PEEK rod systems for lumbar degenerative diseases: clinical experience with a 2-year follow-up. BMC Musculoskelet Disord.

[19] Wangsawatwong P, Sawa AGU, de Andrada Pereira B, Lehrman JN, O’Neill LK, Turner JD, Uribe JS, Kelly BP. Adjacent-segment effects of lumbar cortical screw-rod fixation versus pedicle screw-rod fixation with and without interbody support. J Neurosurg Spine 2021:1–7. 10.3171/2020.11.SPINE20977 34144524

[20] Hirt D, Prentice HA, Harris JE, Paxton EW, Alexander J, Nagasawa DT, Khosla D, Kurtz SM (2021). Do PEEK rods for posterior Instrumented Fusion in the lumbar spine reduce the risk of adjacent segment disease?. Int J Spine Surg.

[21] Kamenova M, Li E, Soleman J, Fiebig O, Mehrkens A, Schaeren S. Posterior stabilization with polyetheretherketone (PEEK) rods and transforaminal lumbar interbody fusion (TLIF) with titanium rods for single-level lumbar spine degenerative disease in patients above 70 years of age. Arch Orthop Trauma Surg 2022. 10.1007/s00402-022-04448-8 PMC10192161 35511354

[22] Qi L, Li M, Zhang S, Xue J, Si H (2013). Comparative effectiveness of PEEK rods versus titanium alloy rods in lumbar fusion: a preliminary report. Acta Neurochir (Wien).

[23] Kurtz SM, Lanman TH, Higgs G, Macdonald DW, Berven SH, Isaza JE, Phillips E, Steinbeck MJ (2013). Retrieval analysis of PEEK rods for posterior fusion and motion preservation. Eur Spine J.

[24] Neukamp M, Roeder C, Veruva SY, MacDonald DW, Kurtz SM, Steinbeck MJ (2015). In vivo compatibility of Dynesys(®) spinal implants: a case series of five retrieved periprosthetic tissue samples and corresponding implants. Eur Spine J.

[25] Shen M, Zhang K, Koettig P, Welch WC, Dawson JM (2011). In vivo biostability of polymeric spine implants: retrieval analyses from a United States investigational device exemption study. Eur Spine J.

