# Supplementary material for: Retrieval analysis of PEEK rods pedicle screw system: three cases analysis
Source: BMC Musculoskelet Disord. 2024 Jun 22;25:488. doi: 10.1186/s12891-024-07600-0 (PMC11193296; doi:10.1186/s12891-024-07600-0)
Supplement: Supplementary file 1 — Supplementary Material 1 [file 12891_2024_7600_MOESM1_ESM.pdf]

Designation: F1717 – 13

Standard Test Methods for Spinal Implant Constructs in a Vertebrectomy Model[1]

This standard is issued under the fixed designation F1717; the number immediately following the designation indicates the year of original adoption or, in the case of revision, the year of last revision. A number in parentheses indicates the year of last reapproval. A superscript epsilon (ε) indicates an editorial change since the last revision or reapproval.

1. Scope

1.1 These test methods cover the materials and methods for the static and fatigue testing of spinal implant assemblies in a vertebrectomy model. The test materials for most combinations of spinal implant components can be specific, depending on the intended spinal location and intended method of application to the spine.

1.2 These test methods are intended to provide a basis for the mechanical comparison among past, present, and future spinal implant assemblies. They allow comparison of spinal implant constructs with different intended spinal locations and methods of application to the spine. These test methods are not intended to define levels of performance, since sufficient knowledge is not available to predict the consequences of the use of a particular device.

1.3 These test methods set out guidelines for load types and methods of applying loads. Methods for three static load types and one fatigue test are defined for the comparative evaluation of spinal implant assemblies.

1.4 These test methods establish guidelines for measuring displacements, determining the yield load, and evaluating the stiffness and strength of the spinal implant assembly.

1.5 Some spinal constructs may not be testable in all test configurations.

1.6 The values stated in SI units are to be regarded as standard. No other units of measurement are included in this standard.

1.7 *This standard does not purport to address all of the safety concerns, if any, associated with its use. It is the responsibility of the user of this standard to establish appropriate safety and health practices and determine the applicability of regulatory limitations prior to use.*

2. Referenced Documents

2.1 *ASTM Standards:*[2]

D638 Test Method for Tensile Properties of Plastics
E4 Practices for Force Verification of Testing Machines
E6 Terminology Relating to Methods of Mechanical Testing
E177 Practice for Use of the Terms Precision and Bias in ASTM Test Methods
E691 Practice for Conducting an Interlaboratory Study to Determine the Precision of a Test Method
E739 Practice for Statistical Analysis of Linear or Linearized Stress-Life (***S-N***) and Strain-Life (ε-***N***) Fatigue Data
E1150 Definitions of Terms Relating to Fatigue (Withdrawn 1996)[3]
F1582 Terminology Relating to Spinal Implants
F2077 Test Methods For Intervertebral Body Fusion Devices

3. Terminology

3.1 *Definitions:*

3.1.1 For definitions of terms relating to these test methods, see Terminology E6, Terminology F1582, and Definitions E1150.

3.2 *Definitions of Terms Specific to This Standard:*

3.2.1 *active length of the longitudinal element*—the straight line distance between the center of attachment of the superior anchor and the center of attachment of the inferior anchor.

3.2.2 *angular displacement at 2 % offset yield (degrees)*—the angular displacement of a construct measured via the actuator that produces a permanent angular displacement in the *X-Y* plane equal to 0.020 times the torsional aspect ratio (see Point A in Fig. 1).

3.2.3 *block moment arm*—the perpendicular to the applied load between the insertion point of an anchor and the axis of the hinge pin.

3.2.4 *compressive or tensile bending stiffness (N/mm)*—the compressive or tensile bending yield force divided by elastic displacement (see the initial slope of line *BC* in Fig. 1).

[1] These test methods are under the jurisdiction of ASTM Committee F04 on Medical and Surgical Materials and Devices and are the direct responsibility of Subcommittee F04.25 on Spinal Devices.

Current edition approved Feb. 1, 2013. Published March 2013. Originally approved in 1996. Last previous edition approved in 2012 as F1717 – 12a. DOI: 10.1520/F1717-13.

[2] For referenced ASTM standards, visit the ASTM website, www.astm.org, or contact ASTM Customer Service at service@astm.org. For *Annual Book of ASTM Standards* volume information, refer to the standard's Document Summary page on the ASTM website.

[3] The last approved version of this historical standard is referenced on www.astm.org.

Copyright © ASTM International, 100 Barr Harbor Drive, PO Box C700, West Conshohocken, PA 19428-2959. United States

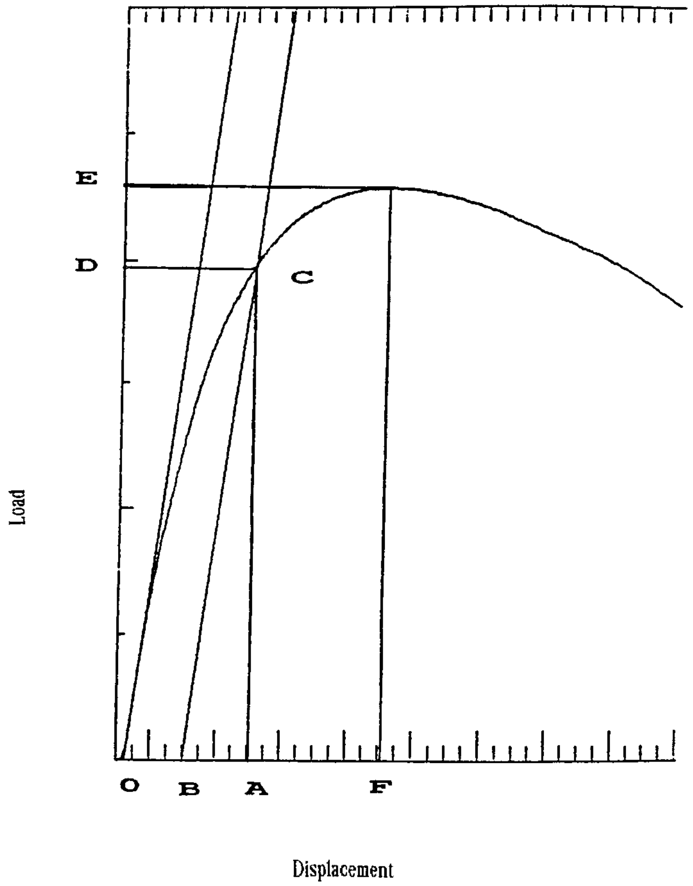

FIG. 1 Typical Load Displacement Curve or Torque Angulation Curve

3.2.5 *compressive or tensile bending ultimate load (N)*—the maximum compressive or tensile force in the *X-Z* plane applied to a spinal implant assembly (see the force at Point E in Fig. 1). The ultimate load should be a function of the device and not of the load cell or testing machine.

3.2.6 *compressive or tensile bending yield load (N)*—the compressive or tensile bending force in the *X-Z* plane necessary to produce a permanent deformation equal to 0.020 times the active length of the longitudinal element (see the force at Point D in Fig. 1).

3.2.7 *coordinate system/axes*—three orthogonal axes are defined in Fig. 2 and Fig. 3. The anterior-posterior axis is *X* with positive being anterior. The medial-lateral axis is *Y* with left being positive when viewed posteriorly. The superior-inferior axis is *Z* with superior being positive.

3.2.8 *displacement at 2 % offset yield (mm)*—the displacement of a construct measured via the actuator that produces a permanent deformation equal to 0.020 times the active length of the longitudinal element (see Point A in Fig. 1).

3.2.9 *elastic angular displacement (degrees)*—the angular displacement at 2 % offset yield (see Point A in Fig. 1) minus the 2 % offset angular displacement (see Point B in Fig. 1). (The distance between Point A and Point B in Fig. 1.)

3.2.10 *elastic displacement (mm)*—the displacement at 2 % offset yield (see Point A in Fig. 1) minus the 2 % offset displacement (see Point B in Fig. 1). (The distance between Point A and Point B in Fig. 1.)

3.2.11 *failure*—permanent deformation resulting from fracture, plastic deformation, or loosening beyond the ultimate displacement or loosening that renders the spinal implant assembly ineffective or unable to adequately resist load.

3.2.12 *fatigue life*—the number of loading cycles, *N*, of a specified character that the spinal implant assembly sustains before failure of a specified nature occurs (see Definitions E1150).

3.2.13 *insertion point of an anchor*—the location where the anchor is attached to the test block. The insertion points shown in Figs. 2-15 are to be adhered to if possible. In situations where the design of the spinal implant assembly or the manufacturer's surgical instructions for installation dictate otherwise, the attachment points may deviate from these dimensions.

3.2.14 *intended method of application*—spinal implant assemblies contain different types of anchors. Each type of anchor has an intended method of application to the spine.

3.2.15 *intended spinal location*—the anatomic region of the spine intended for the application of the spinal implant assembly. Spinal implant assemblies are developed for specific spinal locations such as the anterior cervical spine or the posterior thoracolumbar, lumbar, and lumbosacral spine.

3.2.16 *hinge pin*—the cylindrical rod connecting a test block to a side support. A cervical construct is secured with a 9.6 mm diameter pin and the thoracolumbar, lumbar, and lumbosacral construct uses a 12.7 mm diameter pin.

3.2.17 *longitudinal direction*—the initial spatial orientation parallel to the longitudinal element of the spinal implant assembly. The longitudinal direction is generally in the superior-inferior direction and, therefore, generally parallel to the *z* axis.

3.2.18 *maximum run-out load*—the maximum load that can be applied to a spinal implant assembly where all of the tested constructs have withstood 5 000 000 cycles without a failure.

3.2.19 *permanent deformation*—the displacement (mm) or angular displacement (degree) of the spinal implant construct relative to the initial unloaded condition as measured via the actuator after the applied load, moment, or torque has been removed.

3.2.20 *spinal implant assembly*—a complete spinal implant configuration as intended for surgical use. A spinal implant assembly will contain anchors, interconnections, and longitudinal elements and may contain transverse elements (see Fig. 4, Fig. 6, Fig. 8, Fig. 10, Fig. 12, and Fig. 14).

3.2.21 *spinal implant construct*—a complete spinal implant assembly attached to the appropriate test blocks.

3.2.22 *test block*—the component of the test apparatus for mounting the spinal implant assembly. A specific design of test block is required for each intended spinal location and intended method of application. Fig. 5, Fig. 7, Fig. 9, Fig. 11, Fig. 13, and Fig. 15 describe the recommended designs for the test

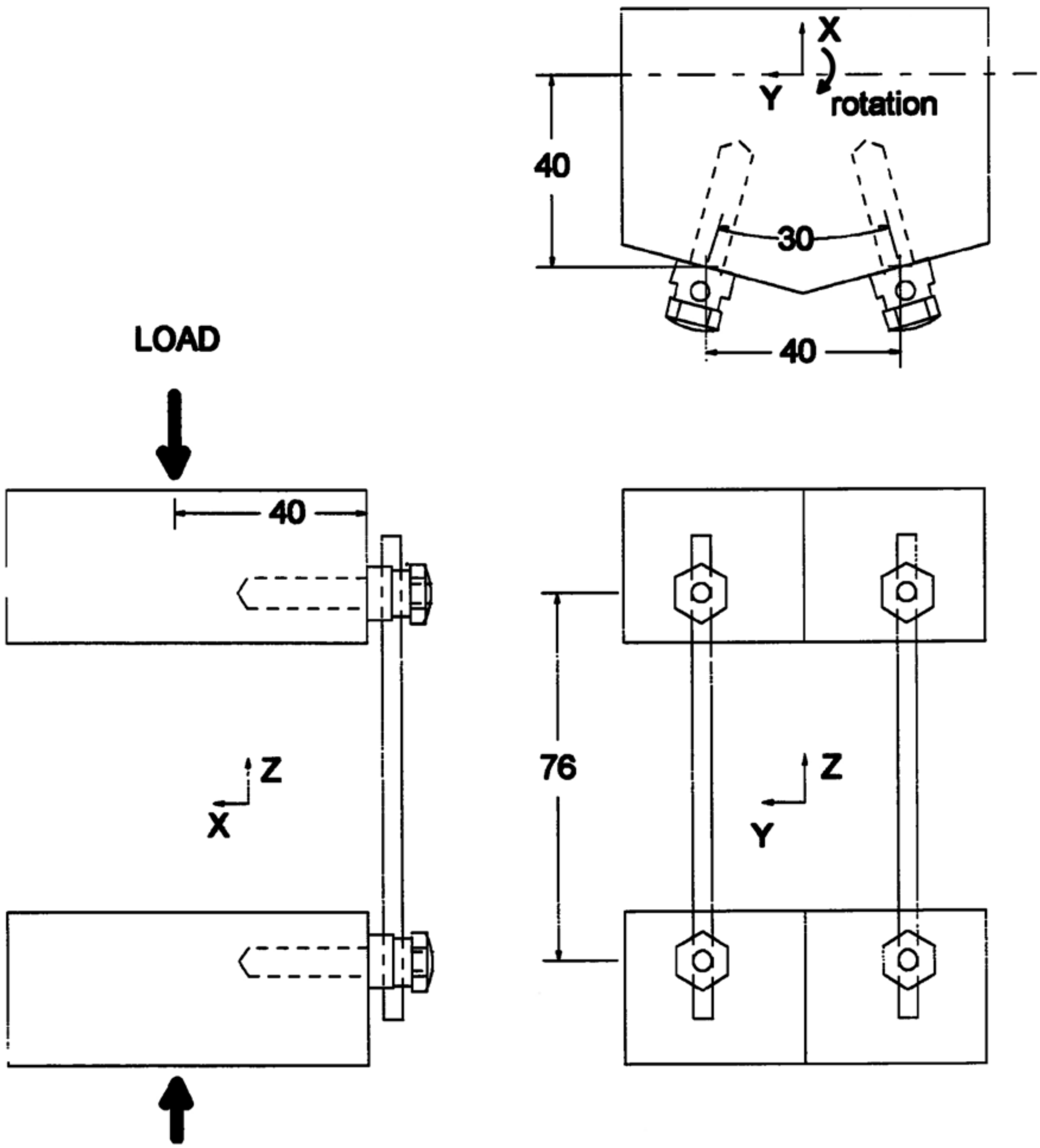

FIG. 2 A Standard Bilateral Construct Containing Screw, Rod and Screw

blocks; however, alternate designs can be used as long as equivalent performance is demonstrated.

3.2.23 *test block load point*—the location on the test block at which the resultant load is transmitted from the test apparatus.

3.2.24 *tightening torque*—the specified torque that is applied to the various threaded fasteners of the spinal implant assembly.

3.2.25 *torsional aspect ratio*—the active length of the longitudinal element divided by the distance from the center of rotation to the insertion point of an anchor (for example: in Fig. 2 1.70 for a 76-mm active length, $X = 40$ mm and $Y = 40/2$ mm).

$$A = \frac{L}{D} = \frac{L}{(x^2 + y^2)^{1/2}} \quad (1)$$

where:

A = torsional aspect ratio,
L = active length of longitudinal element,
D = distance to insertion point,
x = x distance to insertion point, and
y = y distance to insertion point.

3.2.26 *torsional stiffness (N-m/degree)*—the yield torque (N-m) divided by elastic angular displacement (degrees) (the initial slope of line BC in Fig. 1).

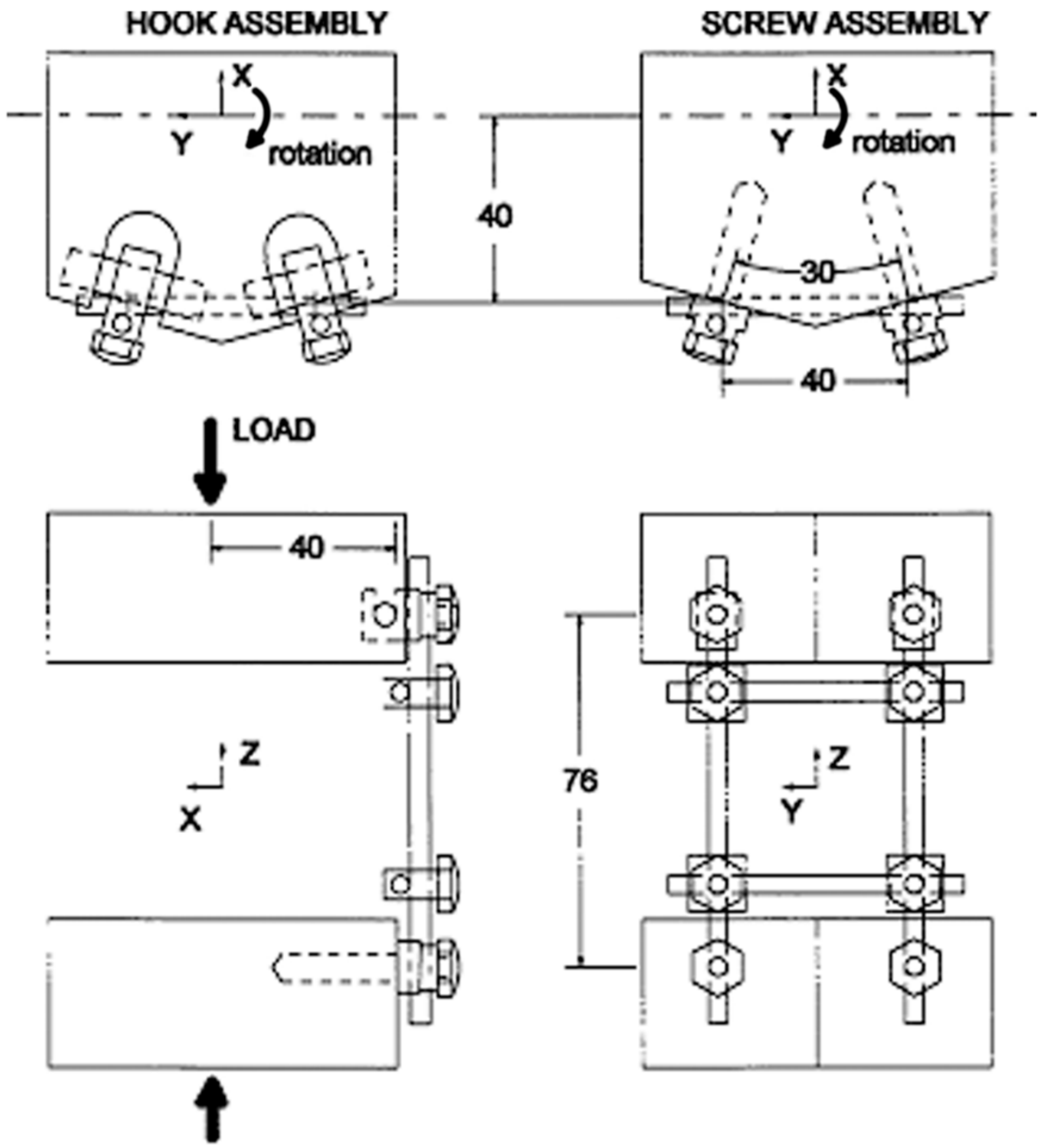

FIG. 3 A Bilateral Hook, Rod, Screw, and Transverse Element Construct

3.2.27 *torsional ultimate load (N-m)*—the maximum torque in the *X-Y* plane applied to a spinal implant assembly (the torque at Point E in Fig. 1). The ultimate torque should be a function of the device and not of the load cell or testing machine.

3.2.28 *two percent (2 %) offset angular displacement (degrees)*—a permanent angular displacement in the *X-Y* plane measured via the actuator equal to 0.020 times the torsional aspect ratio (for example: 1.95° for 1.70 × 0.02 × 180°/pi) (see Point B in Fig. 1).

3.2.29 *two percent (2 %) offset displacement (mm)*—a permanent deformation measured via the actuator equal to 0.020 times the active length of the longitudinal element (for example: 1.52 mm for a 76 mm active length of the longitudinal element or 0.70 mm for 35 mm) (see Point B in Fig. 1).

3.2.30 *ultimate displacement (mm)*—the displacement associated with the ultimate load, ultimate bending load or ultimate torque (the displacement at Point F in Fig. 1).

3.2.31 *yield torque (N-m)*—the torque in the *X-Y* plane required to produce a permanent displacement of 0.020 times the torsional aspect ratio (the torque at Point D in Fig. 1).

3.2.32 *zero displacement intercept (mm)*—the intersection of the straight line section of the load displacement curve and the zero load axis (the zero displacement reference Point 0 in Fig. 1).

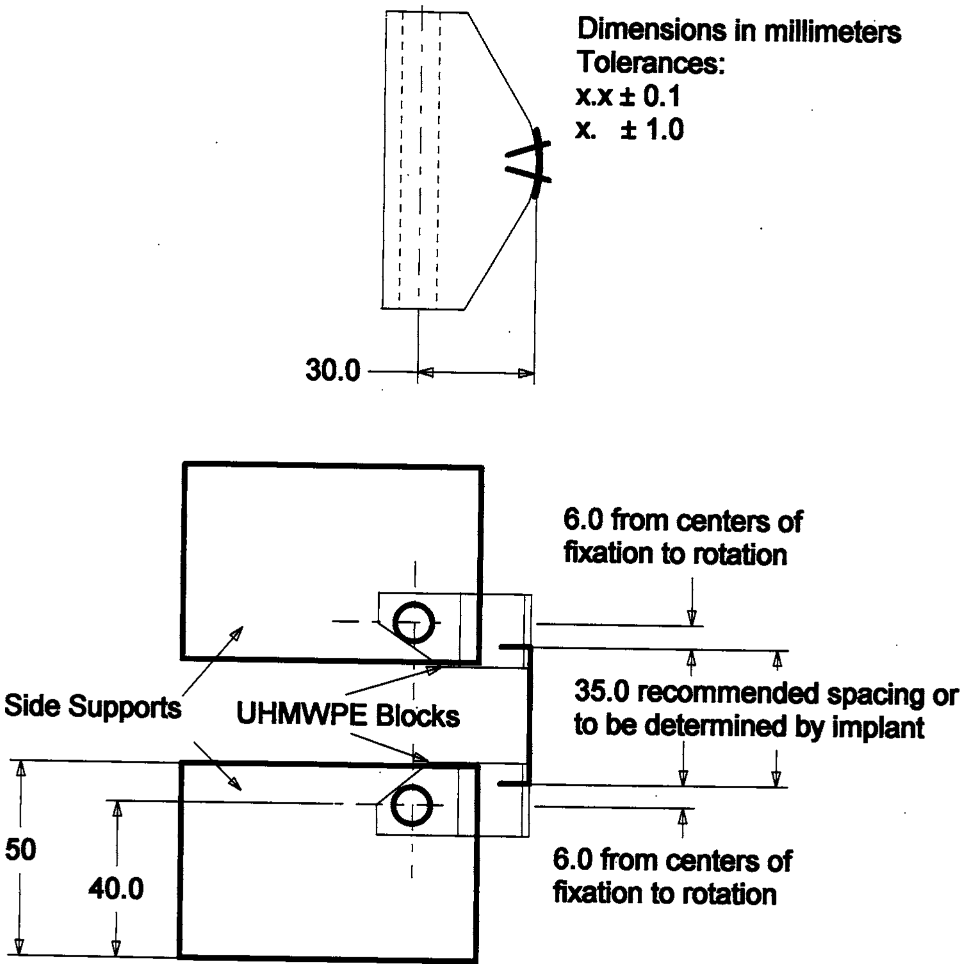

FIG. 4 Cervical Unilateral Construct Test Setup for Screws or Bolts

4. Summary of Test Methods

4.1 Similar test methods are proposed for the mechanical evaluation of cervical spinal implant assemblies (see Fig. 4, Fig. 6, and Fig. 8) and thoracolumbar, lumbar, and lumbosacral spinal implant assemblies (see Fig. 10, Fig. 12, and Fig. 14).

4.2 Testing of the spinal implant assemblies will simulate a vertebrectomy model via a large gap between two Ultra High Molecular Weight Polyethylene (UHMWPE) test blocks. The UHMWPE used to manufacture the test blocks should have a tensile breaking strength equal to 40 ± 3 MPa (see Specification D638). The UHMWPE test blocks (see Fig. 5, Fig. 7, Fig. 9, Fig. 11, Fig. 13, and Fig. 15) will eliminate the effects of the variability of bone properties and morphometry. Alternate designs of test blocks may be used as long as equivalent performance is demonstrated.

4.3 Three static mechanical tests and one dynamic test will evaluate the spinal implant assemblies. The three static mechanical tests are compression bending, tensile bending, and torsion. The dynamic test is a compression bending fatigue test. It is the responsibility of the user of this standard to determine which test(s) is (are) most appropriate for a particular spinal implant assembly.

4.4 A specific clinical indication generally requires a specific spinal implant assembly. Spinal implant assemblies will be evaluated with test configurations which simulate the clinical requirements for the intended spinal location. The intended spinal locations are both anterior (see Fig. 4) and posterior (see Fig. 6 and Fig. 8) surfaces of the cervical spine or both anterior (see Fig. 10) and posterior (see Fig. 12 and Fig. 14) surfaces of the thoracolumbar, lumbar, and lumbosacral spine. The block moment arm (see 6.6) for a test configuration depends on the intended spinal location. The cervical spine configuration (see Fig. 5, Fig. 7, and Fig. 9) specifies one block moment arm, while a larger block moment arm (see Fig. 11, Fig. 13, and Fig. 15) is specified for the thoracolumbar, lumbar, and lumbosacral spine.

4.5 The intended method of application of the spinal implant assembly may vary for specific anatomic regions and clinical indications. Spinal implant assemblies contain different types of anchors. Each type of anchor has an intended method of application to the spine. For example, one assembly may include anterior vertebral body screws and rods (see Fig. 2), while another assembly may contain posterior sacral screws, hooks, rods, and transverse elements (see Fig. 3). The block

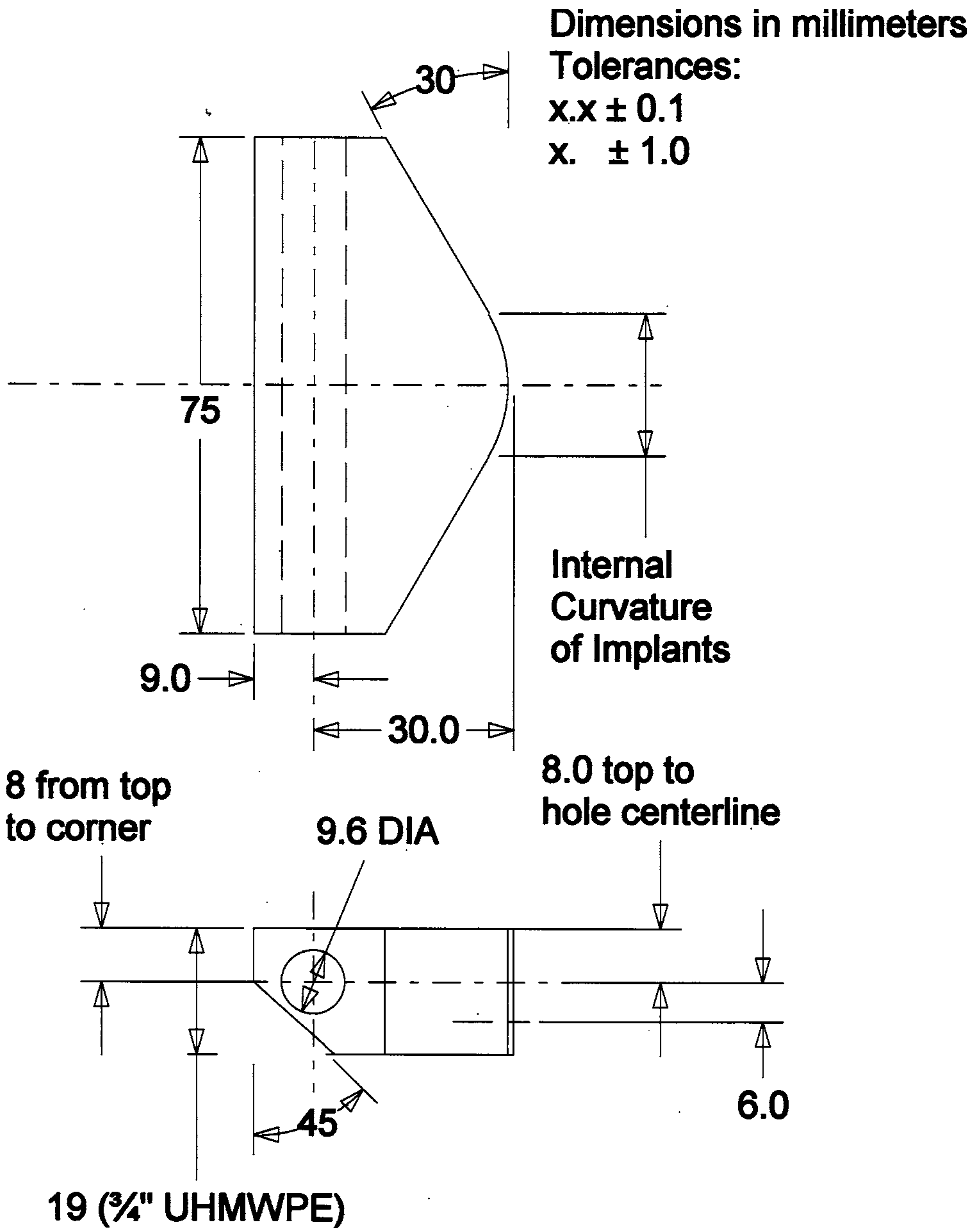

FIG. 5 Cervical Unilateral UHWMPE Block for Screws or Bolts

moment arm of a test configuration will be independent of the intended method of application of a spinal implant assembly; therefore, the test data for different intended methods of application may be compared.

5. Significance and Use

5.1 Spinal implants are generally composed of several components which, when connected together, form a spinal implant assembly. Spinal implant assemblies are designed to provide some stability to the spine while arthrodesis takes place. These test methods outline standard materials and methods for the evaluation of different spinal implant assemblies so that comparison between different designs may be facilitated.

5.2 These test methods are used to quantify the static and dynamic mechanical characteristics of different designs of spinal implant assemblies. The mechanical tests are conducted *in vitro* using simplified load schemes and do not attempt to mimic the complex loads of the spine.

5.3 The loads applied to the spinal implant assemblies *in vivo* will, in general, differ from the loading configurations used in these test methods. The results obtained here cannot be used directly to predict *in vivo* performance. The results can be used to compare different component designs in terms of the relative mechanical parameters.

5.4 Fatigue testing in a simulated body fluid or saline may cause fretting, corrosion, or lubricate the interconnections and

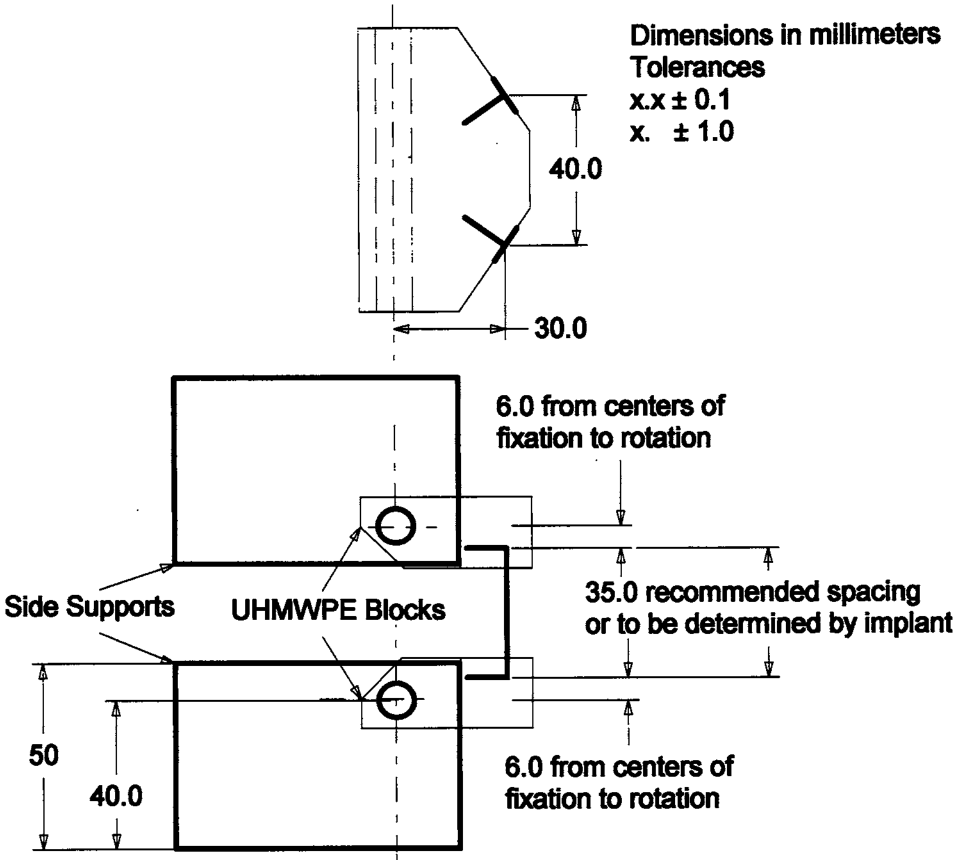

FIG. 6 Cervical Bilateral Construct Test Setup for Screws or Bolts

thereby affect the relative performance of tested devices. This test should be initially performed dry (ambient room conditions) for consistency. The effect of environment may be significant. Repeating all or part of these test methods in simulated body fluid, saline (9 g NaCl per 1000 mL water), a saline drip, water, or a lubricant should be considered. The maximum recommended frequency for this type of cyclic testing should be 5 Hz.

5.5 The location of the longitudinal elements is determined by where the anchors are clinically placed against bony structures. The perpendicular distance to the load direction (block moment arm) between the axis of a hinge pin and the anchor's attachment-points to a UHMWPE block is independent of anchor-type. The distance between the anchor's attachment point to the UHMWPE block and the center of the longitudinal element is a function of the interface design between the screw, hook, wire, cable, and so forth, and the rod, plate, and so forth.

5.6 During static torsion testing, the rotation direction (clockwise or counter clockwise) may have an impact on the results.

6. Apparatus

6.1 Test machines will conform to the requirements of Practices E4.

6.2 The test apparatus allows multiple loading regimes to be applied to all forms of spinal implant assemblies. Two pairs of side supports are mounted on the test machine (see Fig. 4, Fig. 6, Fig. 8, Fig. 10, Fig. 12, and Fig. 14). One pair of side supports attach to the actuator and the second to the load cell. A mounting plate for one of the sets of side support plates should be free to rotate about the *Z* axis for the compression bending, tension bending and fatigue tests. UHMWPE blocks are connected to the side supports via hinge pins. All testing will simulate a vertebrectomy model via a large gap between the two UHMWPE blocks. Select the appropriate design of the UHMWPE blocks (see Fig. 5, Fig. 7, Fig. 9, Fig. 11, Fig. 13, and Fig. 15) to facilitate testing of the spinal implant assembly in a manner that simulates the specific clinical indication at the intended spinal location.

6.3 The design of the UHMWPE blocks causes the plane through the spinal implant assemblies to be parallel to the plane (the *Y-Z* plane) through the axes of the hinge pins. Align the

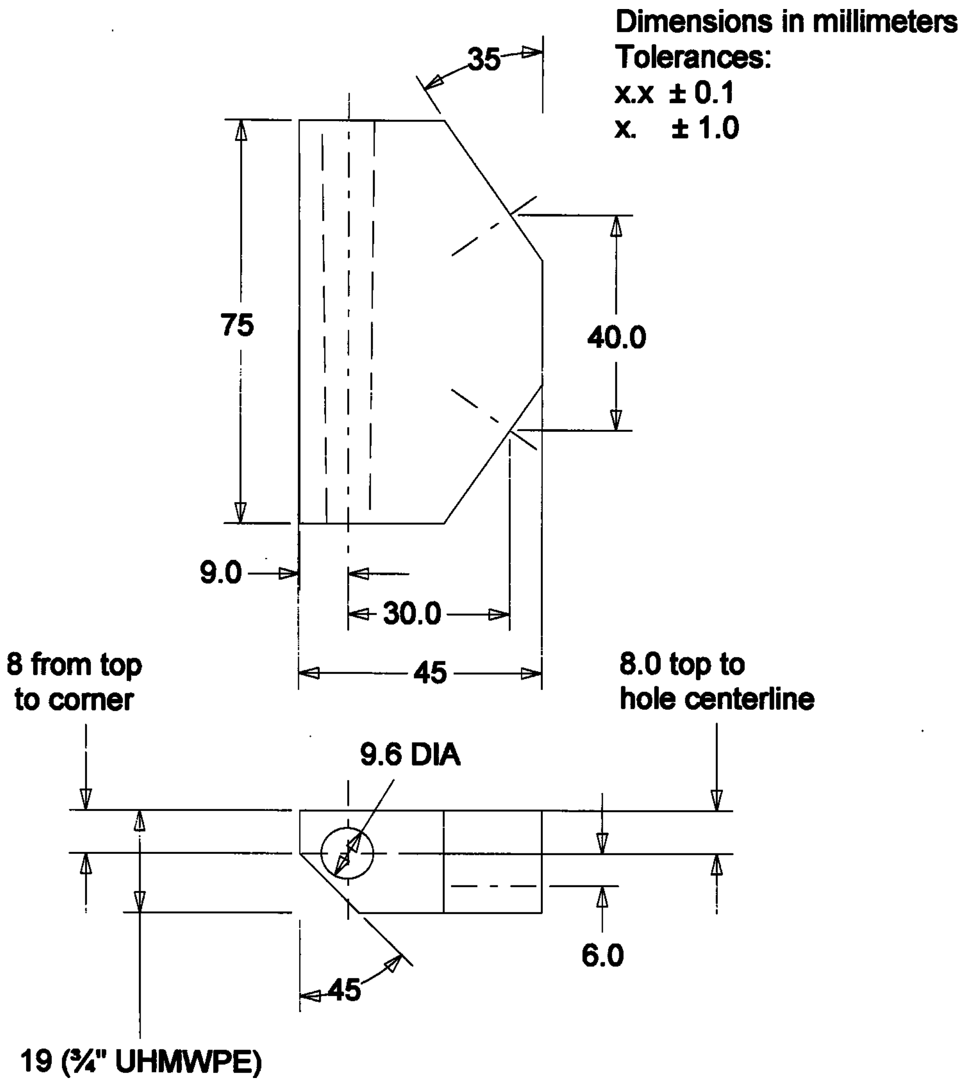

FIG. 7 Cervical Bilateral UHMWPE Block for Screws or Bolts

superior side supports and UHMWPE block with the inferior side supports and UHMWPE block. The center axis of each hinge pin should be perpendicular (±0.5°) to and aligned (±0.5 mm) with the load axis of the test machine. Center the test apparatus in the test machine such that the line through the mid-point (0, 0, Z1) of the inferior hinge pin's axis and the mid-point (0, 0, Z2) of the superior hinge pin's axis is collinear within ±0.1 mm of the load and rotational axis of the test machine's actuator.

6.4 Alternate designs of test blocks may be used as long as equivalence is demonstrated. The solid UHMWPE test blocks may be replaced with metal blocks with UHMWPE inserts of appropriate size. Any surface or component of the spinal assembly which would contact the solid UHMWPE should also contact an appropriate thickness of the UHMWPE. If screws are used to mount the spinal construct to the test blocks (see Fig. 5, Fig. 7, Fig. 11, and Fig. 13), then the screws must be placed into UHMWPE inserts in the alternate design of test block. The diameter of the UHMWPE inserts must be equal to or greater than three times the diameter of the screws.

6.5 If the locations of the superior anchors, inferior anchors, or both sets of anchors are dictated by the longitudinal element and are at different *Z* locations (a diagonal), then the set of anchors should be centered above and below the standard location such that they maintain the average *Z* location. If the anchors are secured into slots in the longitudinal element, then they should be centrally placed in the slots and not at either end to produce a worst-case scenario.

6.6 The distance in the *X* direction between the axis of a hinge pin and the anchors' attachment point should remain constant when comparing spinal implant assemblies. Spinal implant assemblies are designed for two intended spinal locations having two unique block moment arms. The two intended spinal locations are the cervical spinal implant system (see Fig. 4, Fig. 6, and Fig. 8) and the thoracolumbar, lumbar, and lumbosacral spinal implant system (see Fig. 10, Fig. 12, and Fig. 14). The test configurations for the cervical spinal implant system have a block moment arm equal to 30.0 mm. The thoracolumbar, lumbar, and lumbosacral test configurations have a 40-mm block moment arm.

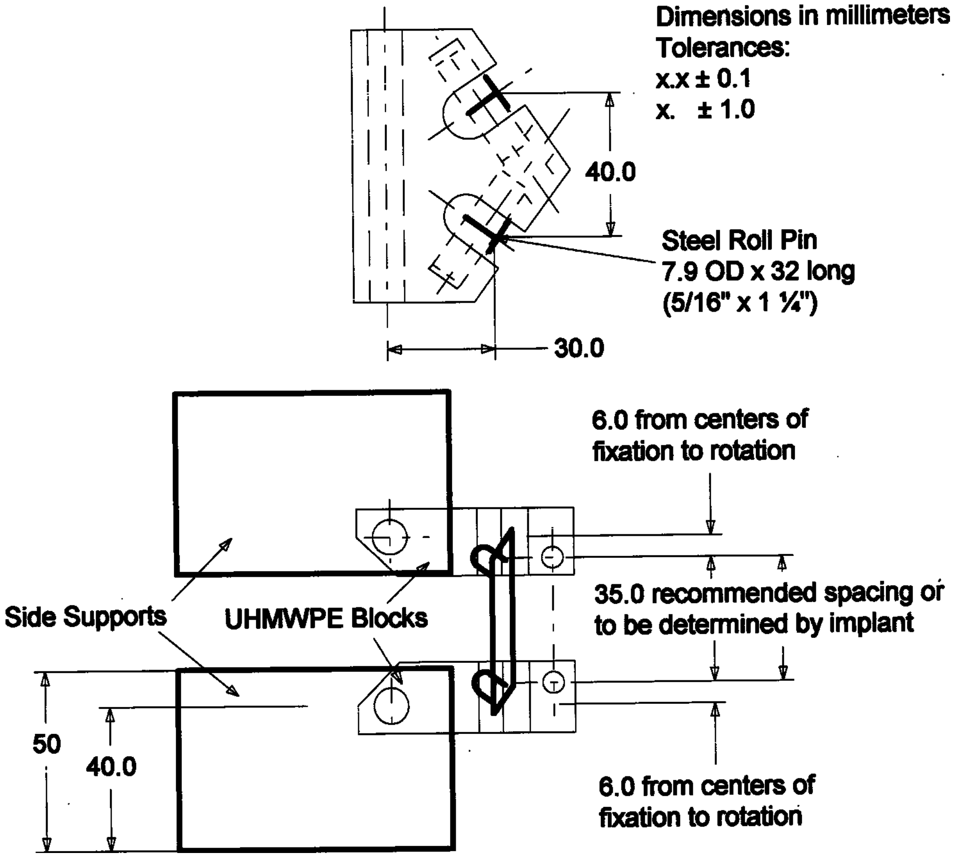

FIG. 8 Cervical Bilateral Construct Test Setup for Hooks, Cables, or Wires

6.7 The UHMWPE blocks have been designed to provide similar block moment arms regardless of the anchor being tested. Different spinal implant assemblies have different intended methods of application to the UHMWPE blocks. The locations of the longitudinal elements are determined by the design of anchors and interconnections. The load capacity of the spinal construct would be a function of the designs of the interconnections, anchors, and longitudinal elements but should not be a function of the test apparatus.

6.8 The hinge pin in the test configuration allows the same test apparatus to be used for the static compression bending test, static tensile bending test, and static torsion test as well as the compression bending fatigue test. The UHMWPE blocks are allowed to rotate around the *Y*-axis of the hinge pin during the compression bending, tensile bending, and fatigue tests.

6.9 Modified bilateral UHMWPE blocks (see Fig. 8, Fig. 9, Fig. 14, and Fig. 15) have been developed for testing hooks, wires, or cables. Steel roll pins are placed into the modified blocks such that the outer surfaces of the roll pins are parallel to the front surfaces of the standard bilateral UHMWPE block (see Fig. 6, Fig. 7, Fig. 12, and Fig. 13). Hooks, wires, and cables are not fully constrained (semi-rigid) fixation devices because they cannot transfer bending moments in the three axes. The combination of the rotation of the modified UHMWPE block on the hinge pin and the rotation of the hooks, wires, or cables around the steel roll pins means that the test configuration would be a mechanism. Therefore, the testing of hooks, wires, and cables necessitates that the modified UHMWPE block must not rotate.

6.10 The relative location (*X* direction versus *Z* direction) between the hinge pin and the insertion point of an anchor produces minimal variation in the block moment arm. The variation in the block moment arm is dependent on the direction of rotation of the UHMWPE blocks. The variation is minimized by having the hinge pins in the UHMWPE blocks rotate past the anchors as the test progresses. Position the hinge pins internal to the anchors during the tension bending test (not shown). Position the hinge pins external to the anchors during the compression bending, torsion and fatigue tests (see Fig. 4, Fig. 6, Fig. 8, Fig. 10, Fig. 12, and Fig. 14).

6.11 The thoracolumbar, lumbar, and lumbosacral test apparatus have a recommended active length of the longitudinal element equal to 76.0 mm and based on the work of Cunningham, et al (**1**).[4] The recommended active length of the longitudinal element for the cervical spinal implant system is 35.0 mm. If the longitudinal element has fixed spacings and the recommended active length cannot be achieved, then select the longitudinal element that is nearest the recommended active length. The active length should be constant for all constructs used in comparative testing.

[4] The boldface numbers in parentheses refer to a list of references at the end of this standard.

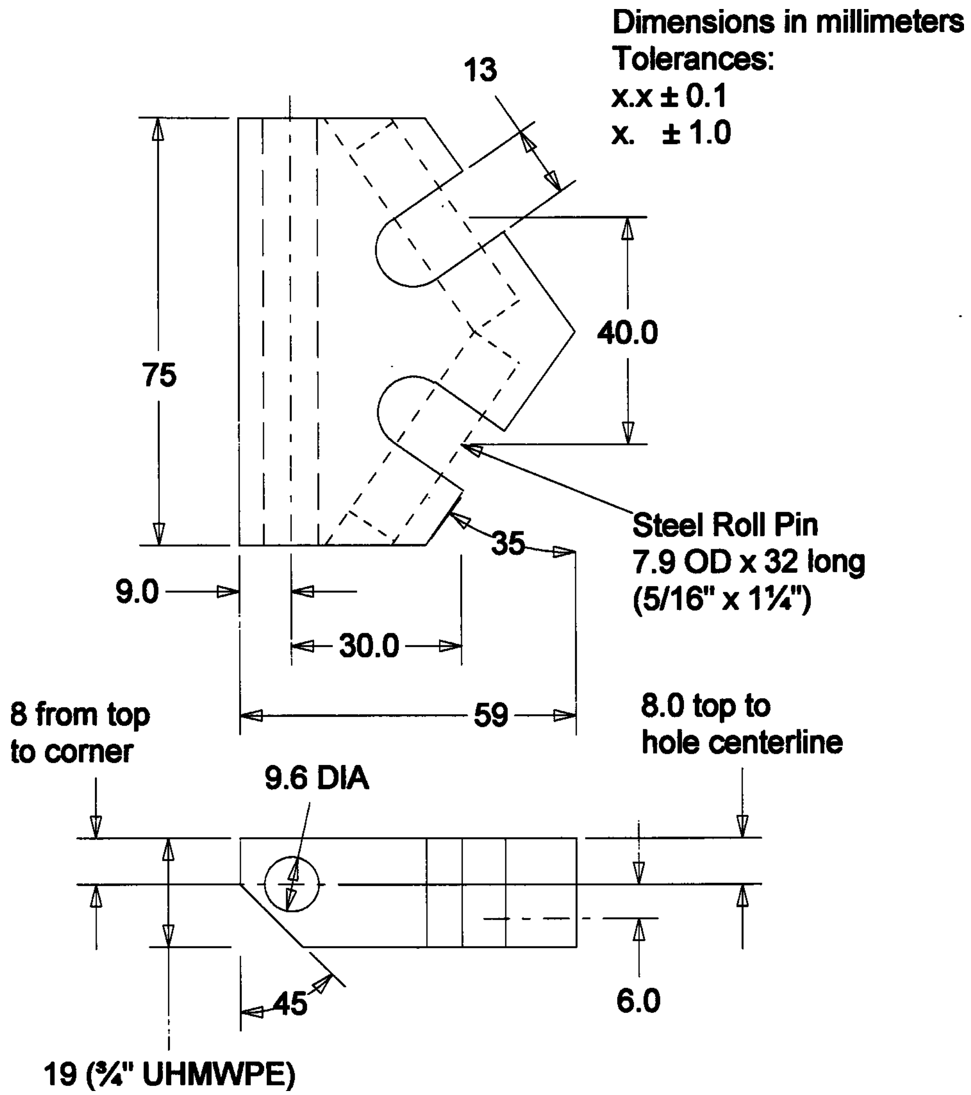

FIG. 9 Cervical Bilateral UHMWPE Block for Hooks, Cables, Wires

6.12 The testing machine and the apparatus used in the static compression bending, static tension bending, and compression bending fatigue tests apply load in the *Z* direction without constraining rotation in the *X-Y* plane. The hinge pin in the apparatus allows rotation in the *X-Z* plane during the static compression bending, static tension bending, and compression bending fatigue tests. The compression bending fatigue test will use the same test configuration as static compressive bending.

6.13 The testing machine or the apparatus used in the static torsion test applies torque about the *Z* axis without constraining displacement in the *Z* direction. Aluminum blocks shall be placed in the apparatus to prevent rotation in the *X-Z* plane during the static torsion tests. The total clearance between an aluminum block, an UHMWPE block, and a base plate will not exceed 0.10 mm.

7. Sampling

7.1 All components in the spinal implant assembly shall be previously unused parts only. Implants shall not be retested.

7.2 Use the UHMWPE test blocks for only one test. The UHMWPE used to manufacture the test blocks should have a tensile breaking strength equal to 40 ± 3 MPa (see Test Method D638). When alternate designs of test blocks are used, then all UHMWPE inserts should be replaced after each test. Alternate designs of test blocks which include steel roll pins (see Fig. 9 and Fig. 15) should replace the steel roll pins and UHMWPE inserts for the hinge after each test.

7.3 Label and maintain the test constructs according to good laboratory practice. Do not disassemble the test construct after testing unless disassembly is necessary to evaluate failure

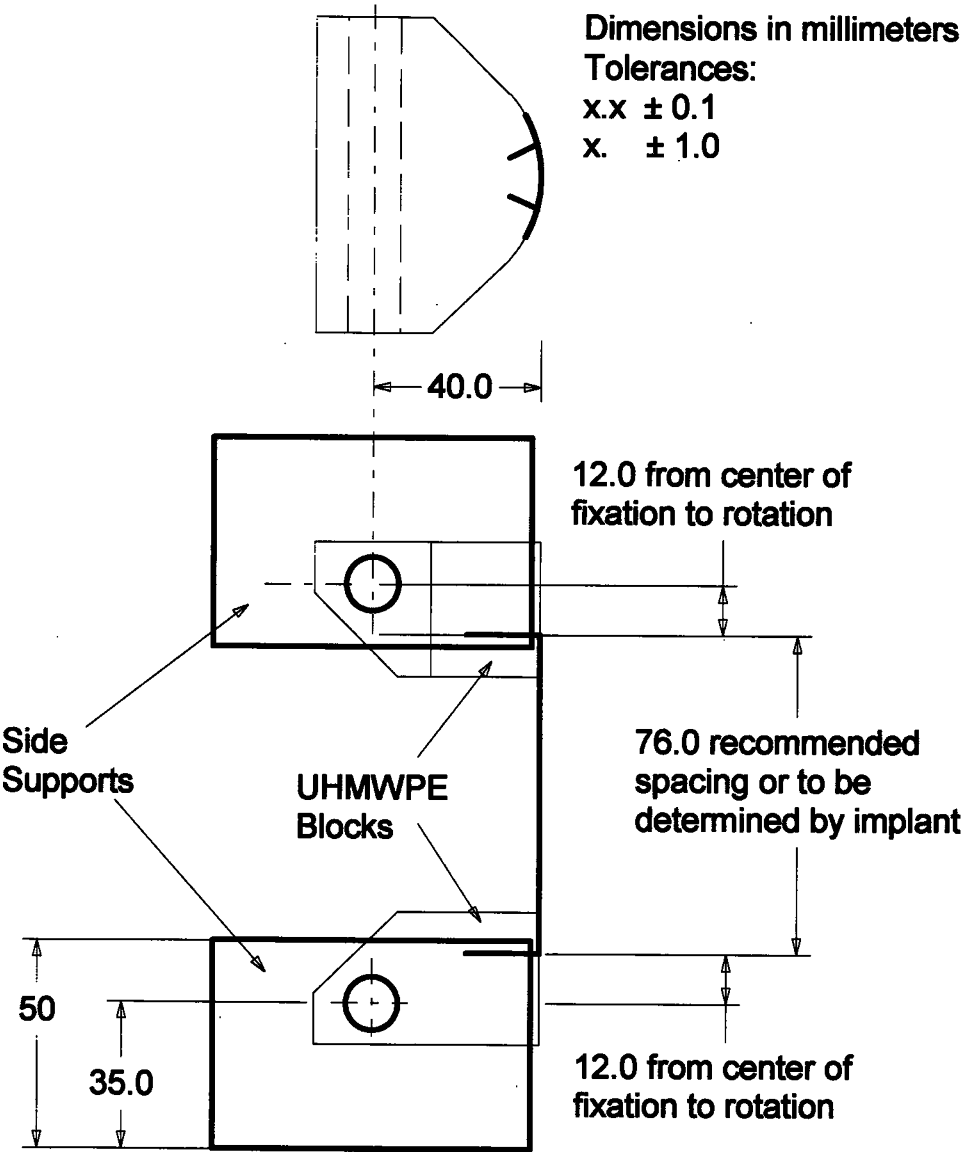

FIG. 10 Lumbar Unilateral UHMWPE Block for Screws or Bolts

surfaces, interconnections, corrosion or loosening surfaces. Photograph the construct prior to disassembly.

7.4 All static tests should have a minimum of five samples. Examination of each load-displacement curve may reveal a laxity in the fixture. After the laxity has been removed, then the initial linear portion of the curve will define the straight line section of the load-displacement curves. The intersection of the straight line section and zero load axis is the zero load displacement (Point 0).

7.5 The results of the fatigue testing will provide a curve of cyclical compression load or compression bending load versus the number of cycles to failure. The suggested initial fatigue loads should be 75, 50, and 25 % of the compression bending ultimate load as determined in the static compression bending test. If a specimen does not fail by 5 000 000 cycles, then testing of that component should be considered run-out. The final sample size is recommended by Practice E739. The differences between the maximum run-out load and a load that results in a failed construct should be less than 10 % of the compression bending ultimate load. Conduct a regression analysis on the load or moment versus number of cycles to failure data.

8. Procedure

8.1 *Procedure for Static Tests*—Evaluate only the load parameters in the relevant direction.

8.1.1 *Static Compression Bending Test:*

8.1.1.1 Select the appropriate UHMWPE blocks for the spinal implant assembly as previously described.

8.1.1.2 Install the anchors according to the manufacturer's instructions. When used as part of a rod/screw construct, variable axis screw anchors shall be inserted in the UHMWPE blocks in a manner that prevents the impingement of any potentially pivoting or rotating features of the anchor against the test block. This may be achieved by inserting the anchor such that, at full angulation of any of the potentially pivoting or

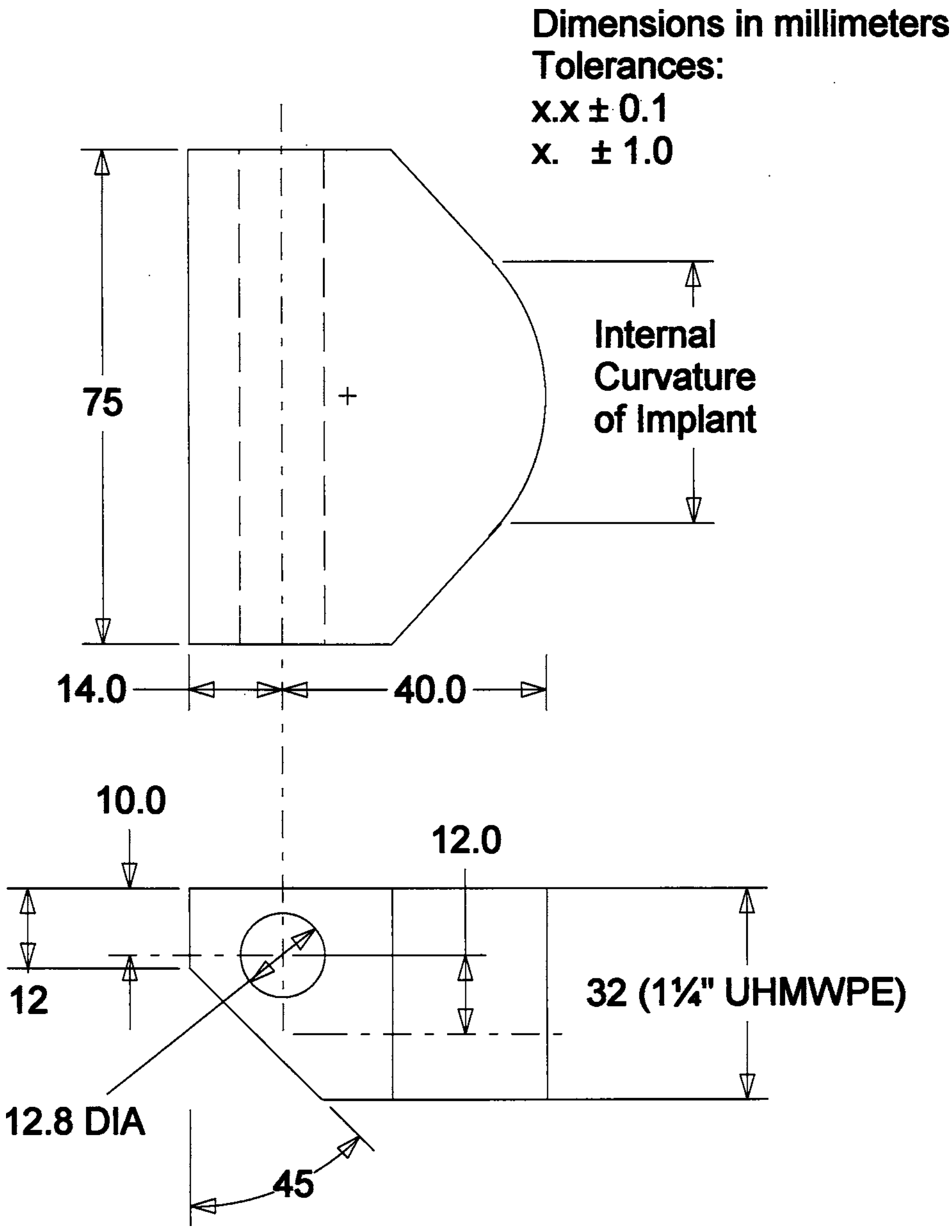

FIG. 11 Lumbar Unilateral UHMWPE Block for Screws or Bolts

rotating features, clearance is always maintained with respect to the test block. If one modified bilateral UHMWPE block is used then place an aluminum spacer block between the modified UHMWPE block and the base plate to stop rotation around the hinge pin. A degree of freedom is eliminated in a similar manner to the axial compression test. If the spinal implant assembly requires two sets of modified bilateral UHMWPE blocks and aluminum spacer blocks, then it is equivalent to an axial compression test.

8.1.1.3 Place the UHMWPE blocks into the test apparatus such that the position of the hinge pins are external to the anchors (the hinge pin in the superior block is more superior than the screw, hook, and so forth). Secure the UHMWPE blocks with hinge pins. If one modified bilateral UHMWPE block is used to test hooks, wires, or cables, then place it superiorly.

8.1.1.4 Complete the spinal implant assembly in a standard construct (see Fig. 4, Fig. 6, Fig. 8, Fig. 10, Fig. 12, and Fig. 14) or a hybrid construct (see Fig. 3). Set the active length of the longitudinal element for the intended spinal location. Apply all tightening, crimping, or locking mechanisms as specified by the manufacturer.

8.1.1.5 Load the test apparatus at a rate up to a maximum of 25 mm/min.

8.1.1.6 Record the load displacement curves. Establish the displacement at 2 % offset yield (mm), elastic displacement (mm), compressive bending yield load *(N)*, compressive bending stiffness (*N*/mm), compressive bending ultimate displacement (mm) and compressive bending ultimate load *(N)*.

8.1.2 *Static Tension Bending Test:*

8.1.2.1 Select the appropriate UHMWPE blocks for the spinal implant assembly as previously described.

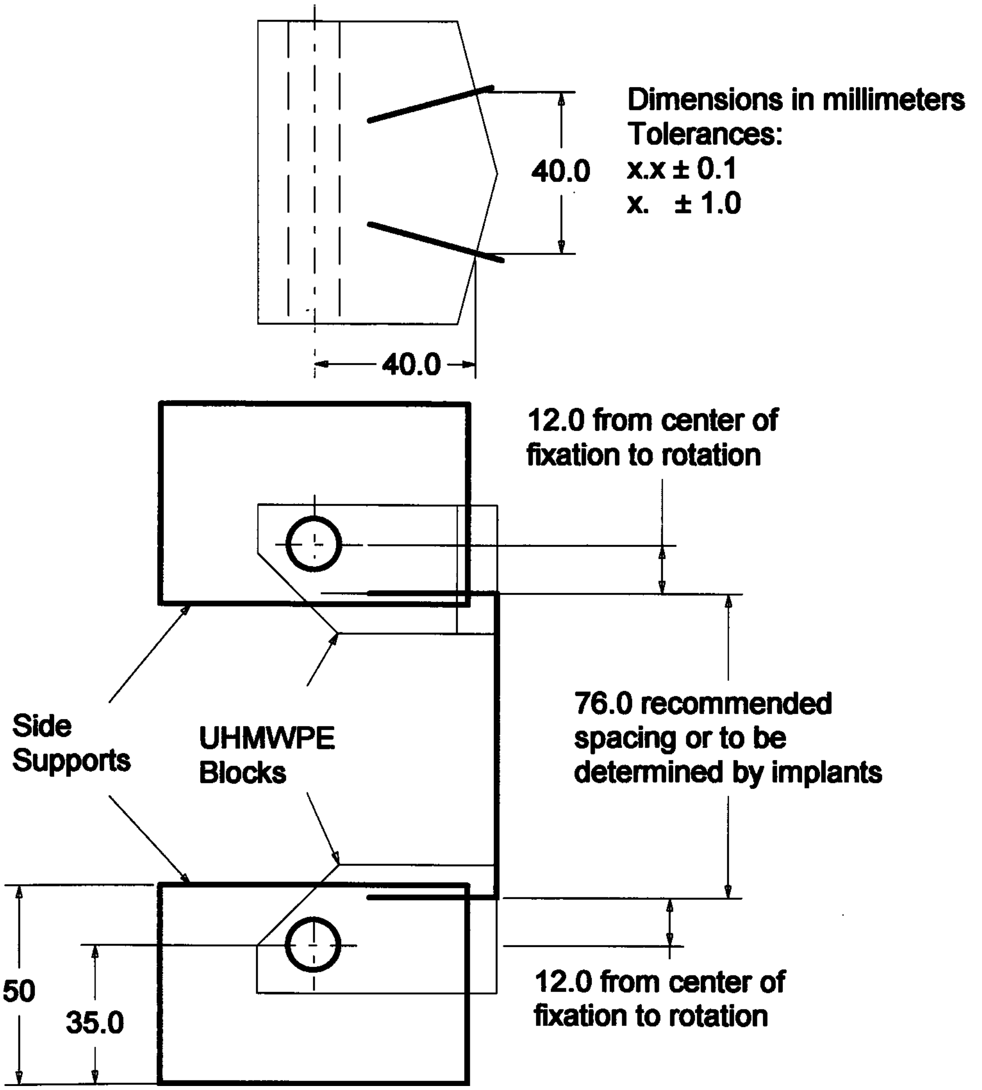

FIG. 12 Lumbar Bilateral Construct Test Setup for Screws

8.1.2.2 Install the anchors according to the manufacturer's instructions. When used as part of a rod/screw construct, variable axis screw anchors shall be inserted in the UHMWPE blocks in a manner that prevents the impingement of any potentially pivoting or rotating features of the anchor against the test block. This may be achieved by inserting the anchor such that, at full angulation of any of the potentially pivoting or rotating features, clearance is always maintained with respect to the test block. If one modified bilateral UHMWPE block is used, then place an aluminum spacer block between the modified UHMWPE block and the base plate to stop rotation around the hinge pin. A degree of freedom is eliminated in a similar manner to the axial compression test. If the spinal implant assembly requires two sets of modified bilateral UHMWPE blocks and aluminum spacer blocks, then it is equivalent to an axial tension test.

8.1.2.3 Place the UHMWPE blocks into the test apparatus such that the position of the hinge pins are internal to the anchors (the hinge pin in the superior block is more inferior than the screw, hook, and so forth). Secure the UHMWPE blocks with hinge pins. If one modified bilateral UHMWPE block is used to test hooks, wires, or cables, then place it superiorly.

8.1.2.4 Complete the spinal implant assembly in a standard construct (see Fig. 4, Fig. 6, Fig. 8, Fig. 10, Fig. 12, and Fig. 14 except the UHMWPE blocks are inverted) or a hybrid construct (see Fig. 3 except the UHMWPE block are inverted). Set the active length of the longitudinal element for the intended spinal location. Apply all tightening, crimping, or locking mechanisms as specified by the manufacturer.

8.1.2.5 Load the test apparatus at a rate up to a maximum of 25 mm/min.

8.1.2.6 Record the load displacement curves. Establish the displacement at 2 % offset yield (mm), elastic displacement (mm), tensile bending yield load *(N)*, tensile bending stiffness (*N*/mm), tensile bending ultimate displacement (mm) and tensile bending ultimate load *(N)*.

8.1.3 *Static Torsional Test:*

8.1.3.1 Select the appropriate UHMWPE blocks for the spinal implant assembly as previously described.

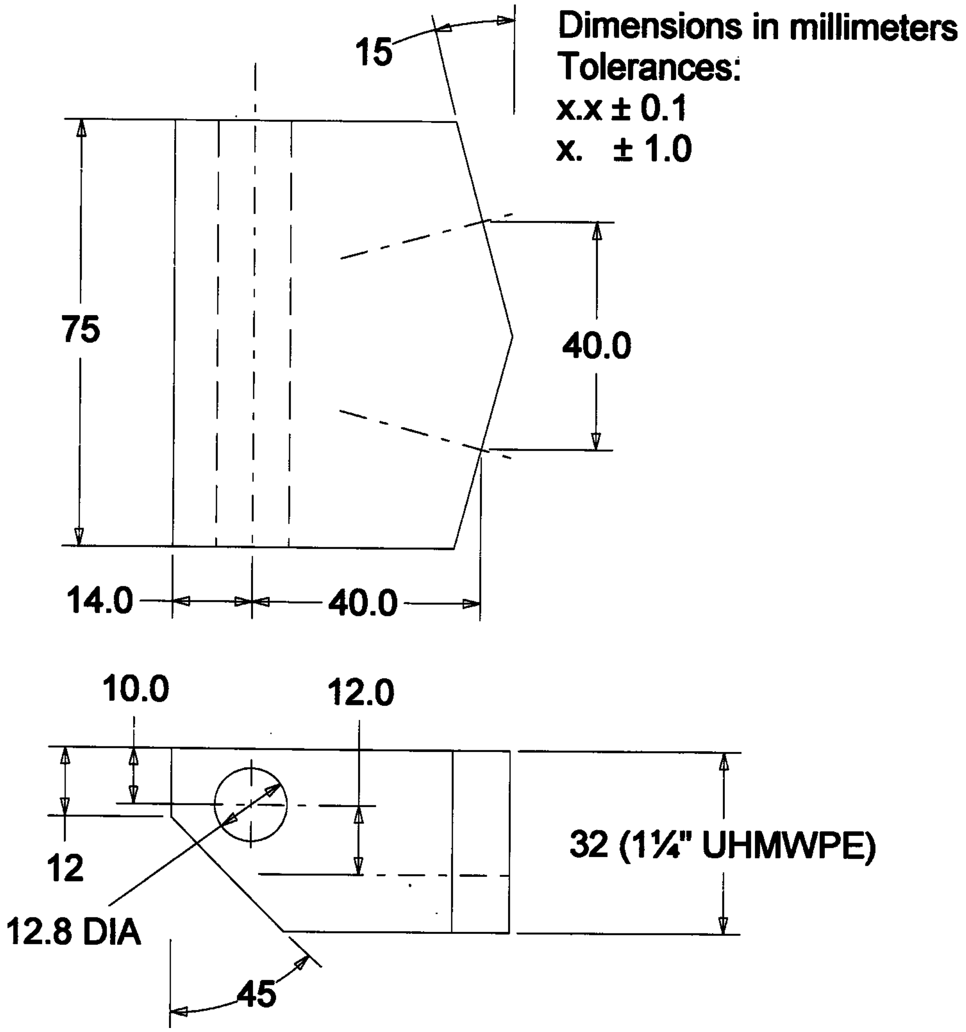

FIG. 13 Lumbar Bilateral UHMWPE Block for Screws or Bolts

8.1.3.2 Install the anchors according to the manufacturer's instructions. When used as part of a rod/screw construct, variable axis screw anchors shall be inserted in the UHMWPE blocks in a manner that prevents the impingement of any potentially pivoting or rotating features of the anchor against the test block. This may be achieved by inserting the anchor such that, at full angulation of any of the potentially pivoting or rotating features, clearance is always maintained with respect to the test block. If the spinal implant assembly contains only hooks, wires, or cables then the system may not be able to resist torsional moments and need not be tested; however, this should be verified by testing.

8.1.3.3 Place the UHMWPE blocks in the test apparatus such that the positions of the hinge pins are external to the anchors. The hinge pin in the superior block is more superior than the screw, hook, and so forth, and the hinge pin in the inferior block is more inferior than the screw, hook, and so forth. Secure the UHMWPE blocks with hinge pins. If only one modified bilateral UHMWPE block is used to test hooks, wires, or cables, then place it superiorly. Attach UHMWPE blocks to the side supports via hinge pins.

8.1.3.4 Place the aluminum blocks between the UHMWPE blocks and the base plates to stop rotation around the hinge pin.

8.1.3.5 Complete the spinal implant assembly in a standard construct (Fig. 4, Fig. 6, Fig. 8, Fig. 10, Fig. 12, and Fig. 14) or a hybrid construct (Fig. 3). Set the active length of the longitudinal element for the intended spinal location. Apply all tightening, crimping, or locking mechanisms as specified by the manufacturer.

8.1.3.6 Load the test apparatus at a maximum rate up to 60°/min. An axial load of approximately zero (*N*) should be maintained during testing.

8.1.3.7 Record the torque-angular displacement curves. Determine the angular displacement at 2 % offset yield (degrees), elastic angular displacement (degrees), yield torque (*N*-m), and torsional stiffness (*N*-m/degree).

8.2 *Procedure for Fatigue Testing:*

8.2.1 Select the appropriate UHMWPE blocks for the spinal implant assembly as previously described. Use unilateral UHMWPE blocks (see Fig. 5 and Fig. 11) for singular longitudinal element constructs. Use bilateral UHMWPE

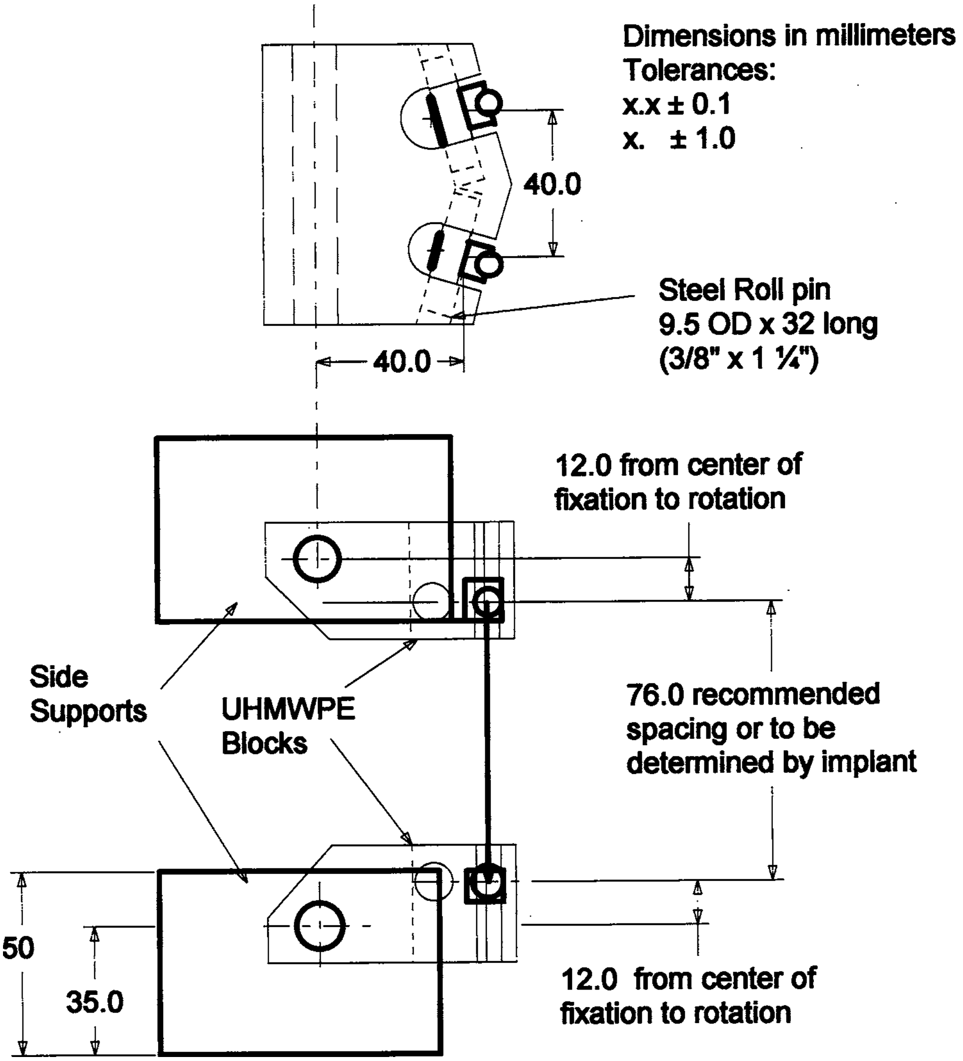

FIG. 14 Lumbar Bilateral Construct Test Setup for Hooks, Cables, or Wires

blocks (see Fig. 7 and Fig. 13) for the testing of screws, bolts, and so forth. Use modified bilateral UHMWPE blocsk (see Fig. 9 and Fig. 15) for the testing of hooks, wires, or cables.

8.2.2 Install the anchors according to the manufacturer's instructions. When used as part a of rod/screw construct, variable axis screw anchors shall be inserted in the UHMWPE blocks in a manner that prevents the impingement of any potentially pivoting or rotating features of the anchor against the test block. This may be achieved by inserting the anchor such that, at full angulation of any of the potentially pivoting or rotating features, clearance is always maintained with respect to the test block. If one modified bilateral UHMWPE block for hooks, wires, cables, and so forth, is used then place an aluminum spacer block between the modified UHMWPE block and the base plate to stop rotation about the hinge pin. The extra degree of freedom is eliminated in a manner similar to the axial compression test. If the spinal implant assembly requires two sets of modified bilateral UHMWPE blocks and aluminum spacer blocks, then the testing mode becomes an axial compression fatigue test.

8.2.3 Place UHMWPE blocks in the test apparatus such that the positions of the hinge pins are external to the anchors. The hinge pin in the superior block is more superior than the anchor and the hinge pin in the inferior block is more inferior than the screw, hook, and so forth. Secure UHMWPE blocks with hinge pins. If only one modified bilateral UHMWPE block is used to test hooks, wires, cables, and so forth, then place it superiorly.

8.2.4 Complete the spinal implant assembly in a standard construct (Fig. 4, Fig. 6, Fig. 8, Fig. 10, Fig. 12, and Fig. 14) or a hybrid construct (Fig. 3). Set the active length of the longitudinal element for the intended spinal location. Apply all tightening, crimping, or locking mechanisms as specified by the manufacturer.

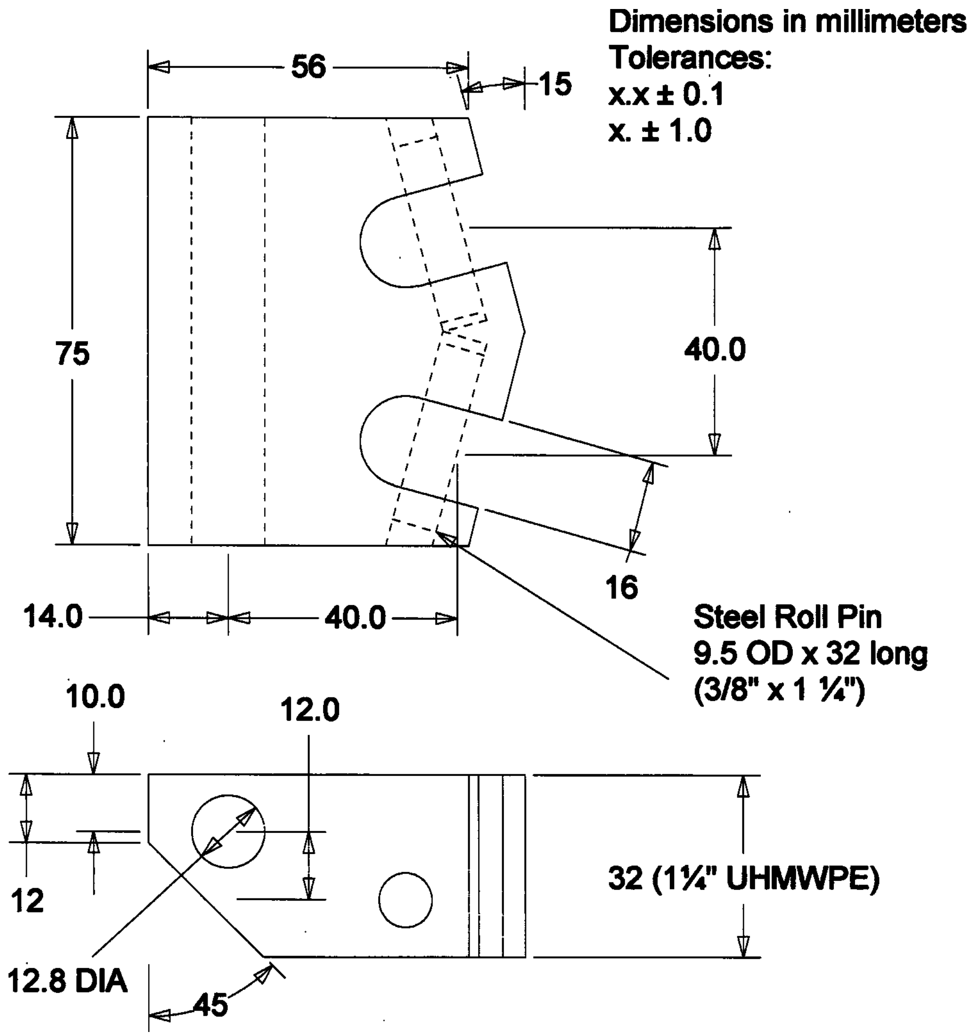

FIG. 15 Lumbar Bilateral UHMWPE Block for Hooks, Cables or Wires

8.2.5 The fatigue test applies a sinusoidal load to the spinal construct. The loading should be maintained via a constant sinusoidal load amplitude control. A constant load ratio (R) for all tests should be established and should be equal to or greater than 10:

$$R = \frac{minimum\ load}{maximum\ load} \geq 10 \quad (2)$$

Example: if minimum load = −200 N and maximum load = −10 N then R = 20. The maximum cycle rate is five cycles per second for the fatigue test. The end of the test occurs when the spinal construct has a failure or reaches run-out.

8.2.6 Evaluate two specimens at the initial fatigue loads. Establish the maximum run-out load (no specimens fail before 5 000 000 cycles). Continue fatigue testing pairs of specimens until the difference between a load in which a construct has failed and the maximum run-out load is no greater than 10 % of the compression bending ultimate load. A semi-log fatigue curve of the compression bending load versus number of cycles at failure will be plotted.

8.2.7 Note the initial and secondary failures, modes of failure, and deformations of components prior to removing the spinal construct from the test apparatus. Evaluate all surface changes.

9. Report

9.1 The report should specify the spinal implant components, the spinal implant assembly, the intended spinal location, and the numbers of specimens tested. Describe all relevant information about the components including name, lot number, manufacturer, material, part number, and so forth. Also include any specific information necessary to produce the assembly, including the tightening torque.

9.2 Include an illustration of the exact loading configurations. Describe the similarities and differences to relevant figures contained herein. Report the active length. Report the block moment arm and the distance in the X direction between the centerline of the longitudinal element and the insertion point of the anchors on the UHMWPE blocks. For constructs using variable axis screw anchors, describe the measure

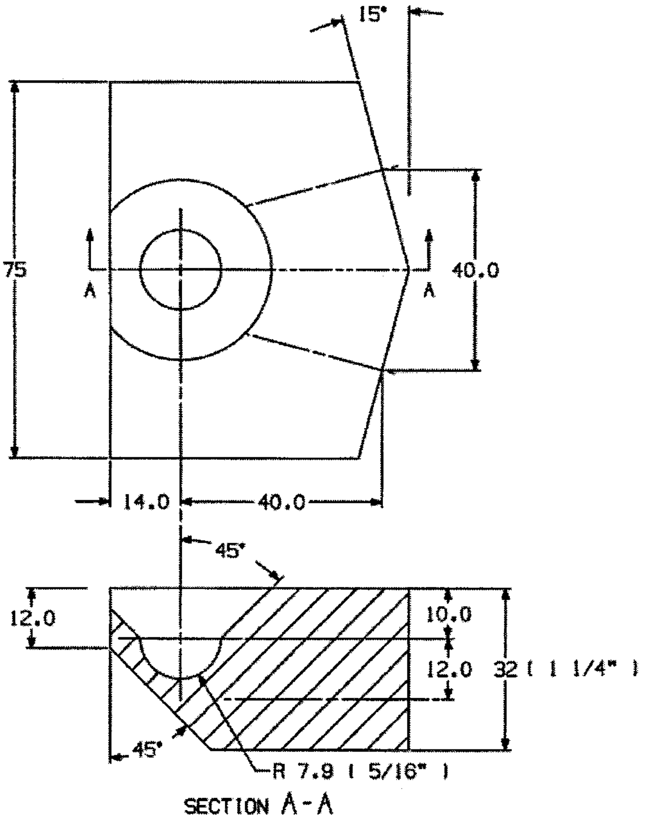

FIG. 16 Alternate Lumbar Bilateral UHMWPE Block for Screws and Bolts

employed to prevent impingement of pivoting or rotating features against the test block. Note any deviations from the recommended test procedure. State the rate of loading.

9.3 The report of the static mechanical testing shall include a complete description of all failures, modes of failure, or deformations of the spinal implant assembly or test apparatus. Include any noticeable fretting or surface texturing. The static mechanical test report shall:

9.3.1 Show the load-displacement curves for all static compression bending tests. Report all static compression bending test data, the mean and standard deviation for the displacement at 2 % offset yield (mm), elastic displacement (mm), compressive bending yield load (*N*), compressive bending stiffness (*N*/mm), compressive bending ultimate displacement (mm), and compressive bending ultimate load (*N*).

9.3.2 Show all load-displacement curves for the static tension bending test. Report all static tension bending test data, the mean and standard deviation for the displacement at 2 % offset yield (mm), elastic displacement (mm), tensile bending yield load (*N*), tensile bending stiffness (*N*/mm), tensile bending ultimate displacement (mm), and tensile bending ultimate load (*N*).

9.3.3 Show the torque-angular displacement curves for all static torsional tests. Report all static torsional test data, the mean and standard deviation for the angular displacement at 2 % offset yield (degrees), elastic angular displacement (degrees), yield torque (*N*-m), and torsional stiffness (*N*-m/degree). Report the direction of rotation for the tests along with the reference point.

9.3.4 Show the reason for each test being halted (for example, device fracture, screws pulling out of UHWMPE blocks, UHWMPE blocks touching, clevis fixtures touching, and so forth).

9.4 The report of the dynamic mechanical testing shall:

9.4.1 State the fatigue test environment, load wave form, and test frequency. State the final sample sizes and load versus number of cycles at failure for all fatigue tests. State the load levels for the specimens enduring 5 000 000 cycles and the maximum run-out load. Report the constant load ratio (*R*).

9.4.2 Report all initial and secondary failures, modes of failure and deformations of components for the spinal implant assembly and the test apparatus. Fatigue failures should include a description of the failure initiation site, propagation zone and ultimate failure zone. Describe all surface changes, any fretting of interfaces or loosening of interconnections. Include pictures of failure surfaces and surface texturing from fretting.

9.4.3 Plot a semi-log fatigue curve of the compression or compression bending load versus number of cycles at failure. Indicate specimens that have not failed before 5 000 000 cycles.

9.4.4 Report a regression analysis of the compression load or compression bending load versus number of cycles for only failed constructs.

10. Precision and Bias

10.1 *Precision*—The precision of these test methods is based on two interlaboratory studies (ILS) conducted in 2008 and 2011. Six laboratories participated in this study. Not all six laboratories were used for the two interlaboratory studies. Each of the six labs was asked to report five replicate test results of a uniform assembly of screws, set screws, rods, and cross connectors, for ten different measurement parameters. Every "test result" reported represents an individual determination. Except for the use of only six laboratories and a single material, Practice E691 was followed for the design and analysis of the data; the details are given in ASTM Research Report No. F04–1012[5] and F04–1013.[6]

10.1.1 *Repeatability Limit (r)*—Two test results obtained within one laboratory shall be judged not equivalent if they differ by more than the "*r*" value for that material; "*r*" is the interval representing the critical difference between two test results for the same material, obtained by the same operator using the same equipment on the same day in the same laboratory.

10.1.1.1 Repeatability limits are listed in Tables 1-10.

10.1.2 *Reproducibility Limit (R)*—Two test results shall be judged not equivalent if they differ by more than the "*R*" value for that material; "*R*" is the interval representing the critical difference between two test results for the same material, obtained by different operators using different equipment in different laboratories.

10.1.2.1 Reproducibility limits are listed in Tables 1-10. Tables 1-6 refer to compressive bending testing and Tables 7-10 refer to torsion testing.

10.1.3 The above terms (repeatability limit and reproducibility limit) are used as specified in Practice E177.

10.1.4 Any judgment in accordance with statements 10.1.1 and 10.1.2 would normally have an approximate 95 % probability of being correct; however, the precision statistics obtained in this ILS must not be treated as exact mathematical quantities which are applicable to all circumstances and uses. The limited number of materials tested and laboratories reporting results guarantees that there will be times when differences greater than predicted by the ILS results will arise, sometimes with considerably greater or smaller frequency than the 95 % probability limit would imply. Consider the repeatability limit and the reproducibility limit as general guides, and the associated probability of 95 % as only a rough indicator of what can be expected.

10.1.5 The explanations for *r* and *R* are intended to present a meaningful way of considering the approximate precision of the test method. The data in Tables 1-10 should not be applied rigorously to acceptance or rejection of material, as those data are specific to this ILS and may not be representative of other lots, materials, surgical applications or laboratories. Users of this test method should apply the principles outlined in Practice E691 to generate data specific to their laboratory and materials.

10.2 *Bias*—At the time of the study, there was no accepted reference material suitable for determining the bias for these test methods, therefore no statement on bias is being made.

10.3 The precision statement was determined through statistical examination of 280 results, from six laboratories, on a single material. The material tested was described as the following:

10.3.1 *Material A*—DePuy Spine Summit 3.5 mm mini polyaxial screws with set screws, 3 mm rods, and cross connectors (for torsion tests only). Product codes may be found in ASTM Research Report No. F04–1012. The material tested was designed for application in the upper thoracic spine and the magnitudes of the mean, standard deviations and resulting repeatability and reproducibility values may be different than materials used in other surgical applications. See, for example, Stanford et al (**5**).

10.3.2 *Material B*—Lanx Ø6.5 mm (Torsion) and Ø7.5 mm (Compression) Monoaxial screws with sets screws and 5.5 mm stainless steel rods. Product codes may be found in Research Report No. F04-1013. The material tested was designed for the thoracolumbar spine and the magnitudes of the mean, standard deviations, and resulting repeatability and reproducibility values may be different than materials used in other surgical applications. See, for example, Stanford et al (**5**) .

11. Keywords

11.1 fatigue test methods; spinal implant assembly; spinal implant construct; static test methods ; vertebrectomy model

[5] Supporting data have been filed at ASTM International Headquarters and may be obtained by requesting Research Report RR:F04-1012.

[6] Supporting data have been filed at ASTM International Headquarters and may be obtained by requesting Research Report RR:F04-1013.

TABLE 1 Compressive Bending Ultimate Displacement (mm)

NOTE 1—The ILS conducted in 2011 was compressed to a target displacement, therefore the *r* and *R* for ultimate displacement is not presented.

Material	Average[A] x	Repeatability Standard Deviation S_r	Reproducibility Standard Deviation S_R	Repeatability Limit r	Reproducibility Limit R
A	61.51	0.88	8.66	2.45	24.25

[A] The average of the laboratories' calculated averages.

TABLE 2 Compressive Bending Ultimate Load (N)

NOTE 1—Material B Ultimate Load represents ultimate load at target displacement.

Material	Average[A] $\bar{x}$	Repeatability Standard Deviation S_r	Reproducibility Standard Deviation S_R	Repeatability Limit r	Reproducibility Limit R
A	193.15	6.49	23.71	18.16	66.39
B	699.86	25.65	34.44	71.83	96.42

[A] The average of the laboratories' calculated averages.

TABLE 3 Compressive Bending Stiffness (N/mm)

Material	Average[A] $\bar{x}$	Repeatability Standard Deviation S_r	Reproducibility Standard Deviation S_R	Repeatability Limit r	Reproducibility Limit R
A	6.90	0.36	0.78	1.02	2.18
B	70.53	1.87	4.29	5.20	12.02

[A] The average of the laboratories' calculated averages.

TABLE 4 Compressive Bending Yield Load (N)

Material	Average[A] $\bar{x}$	Repeatability Standard Deviation S_r	Reproducibility Standard Deviation S_R	Repeatability Limit r	Reproducibility Limit R
A	81.04	8.10	11.03	22.67	30.89
B	495.91	16.42	27.82	45.97	77.90

[A] The average of the laboratories' calculated averages.

TABLE 5 Elastic Displacement (mm)

Material	Average[A] $\bar{x}$	Repeatability Standard Deviation S_r	Reproducibility Standard Deviation S_R	Repeatability Limit r	Reproducibility Limit R
A	11.50	1.72	2.18	4.82	6.11
B	6.81	0.32	0.45	0.89	1.26

[A] The average of the laboratories' calculated averages.

TABLE 6 Displacement at 2 % Offset Yield (mm)

Material	Average[A] $\bar{x}$	Repeatability Standard Deviation S_r	Reproducibility Standard Deviation S_R	Repeatability Limit r	Reproducibility Limit R
A	12.87	1.73	2.04	4.85	5.71
B	8.58	0.32	0.66	0.89	1.86

[A] The average of the laboratories' calculated averages.

TABLE 7 Torsional Stiffness (N-m/degrees)

Material	Average[A] $\bar{x}$	Repeatability Standard Deviation S_r	Reproducibility Standard Deviation S_R	Repeatability Limit r	Reproducibility Limit R
A	0.64	0.06	0.27	0.16	0.75
B	5.32	0.89	1.47	2.48	4.12

[A] The average of the laboratories' calculated averages.

TABLE 8 Yield Torque (N-m)

Material	Average[A] $\bar{x}$	Repeatability Standard Deviation S_r	Reproducibility Standard Deviation S_R	Repeatability Limit r	Reproducibility Limit R
A	3.27	0.33	0.90	0.92	2.51
B	19.55	4.14	4.14	11.58	11.58

[A] The average of the laboratories' calculated averages.

TABLE 9 Elastic Angular Displacement (degrees)

Material	Average[A] $\bar{x}$	Repeatability Standard Deviation S_r	Reproducibility Standard Deviation S_R	Repeatability Limit r	Reproducibility Limit R
A	5.68	0.94	2.47	2.62	6.92
B	3.78	0.43	0.96	1.19	2.70

[A] The average of the laboratories' calculated averages.

TABLE 10 Angular Displacement at 2 % Offset Yield (degrees)

Material	Average[A] $\bar{x}$	Repeatability Standard Deviation S_r	Reproducibility Standard Deviation S_R	Repeatability Limit r	Reproducibility Limit R
A	7.62	1.12	2.57	3.13	7.20
B	5.86	0.47	0.97	1.31	2.72

[A] The average of the laboratories' calculated averages.

APPENDIXES

(Nonmandatory Information)

X1. RATIONALE

X1.1 Spinal implant assemblies contain many different component designs and can be assembled into a wide variety of configurations and combinations for different clinical indications dependent on the clinical requirements, intended clinical location, and intended method of application. The purpose of these test methods is to provide the framework for a comprehensive series of mechanical tests that can be used to compare different implant designs in a consistent manner.

X1.2 A spinal implant assembly contains groups of components necessary for specific clinical indications. These test methods contain test configurations for the evaluation of spinal implant assemblies that simulate the clinical requirements for an intended clinical location and method of application. Some designs of thoracolumbar, lumbar, and lumbosacral spine system are intended for both anterior and posterior attachment. These systems include anterior vertebral body/pedicle/sacral screws, hooks, rods, and transverse interconnections. Fig. 2 is an example of a standard test configuration. Fig. 6 or Fig. 12 are constructs which simulating a common clinical group of components normally applied to the anterior surface of a vertebral body or posterior vertebral structures. The hybrid test configuration seen in Fig. 3 would normally be applied posteriorly and contains sacral screws, hooks, rods, and transverse interconnections.

X1.3 A spinal implant construct installed in the test apparatus will simultaneously evaluate all components within the assembly in the worst case test configuration (vertebrectomy model). A vertebrectomy is assumed to be a worst case

scenario because all loads are transferred from the fixture and are transmitted only through the implant assembly. All proposed test configurations are based on anatomical dimensions. Some asymmetric test assemblies may not be applicable to these test methods. In these cases, the hinge pin might be replaced with a ball and socket.

X1.4 These test methods cover the static and cyclic evaluation of spinal implant assemblies. The purpose of spinal implants is to provide short term stability while arthrodesis takes place. These test methods do not address the long term mechanical stability of spinal implants, nor do they address implants that do not lead to spinal fusion. The fatigue testing in these test methods establish the maximum run out load where all of the tested constructs have withstood 5 000 000 cycles without a failure. 5 000 000 cycles represents the number of loading cycles a specimen might experience within two years based on moderate activity (~7000 cycles per day).

X1.5 Uniaxial torque and combination axial and bending loading are applied in these test methods. Numerous combinations of multiaxial loading conditions *in vivo* have not yet been fully defined. These test methods outline a series of simplistic static and dynamic loading conditions and do not attempt to mimic the complex loading patterns in the spine.

X1.6 The influence of simulated body fluid or saline may affect the relative performance of tested devices. The test methods outlined here should be performed dry (ambient room conditions) to eliminate unwanted complexity resulting from environmental factors. This will reduce the variability of the results. Individual investigators may consider additional evaluations in simulated body fluid, saline, water, or lubricants to address environmental factors. It should be noted that corrosive fatigue testing may be influenced by the cycle rate, therefore the maximum cycle rate should be reduced.

X1.7 The variation in the block moment arm for the static and fatigue bending tests is a function of the relative location (*X*direction) between the hinge pin and the insertion point of the anchor. The variation in the relative location is dependent on the direction of rotation (tension or compression) and arrangement of the UHMWPE blocks. The variation in the block moment arm for the thoracolumbar, lumbar, and lumbosacral test configuration ranges between ±4 % for a 57-mm displacement or 39° rotation of each block. The block moment arm variations for the cervical test configuration range between ±2 % for a 29-mm displacement or 27° rotation of each block.

X1.8 Reporting the compression or tensile bending moments and the bending moments versus numbers of cycles at failure are not recommended because confusion regarding the moment arm may exist. The block moment arms are constants per these test methods. The block moment arm is 30.0 mm for cervical constructs (see Fig. 4, Fig. 6, and Fig. 8) and 40.0 mm for thoracolumbar, lumbar, and lumbosacral constructs (see Fig. 10, Fig. 12, and Fig. 14). However, the moment arm at the longitudinal element can vary from one spinal assembly to another. The moment arm at the longitudinal element is the sum of the block moment arm and the *X*distance from the insertion point of an anchor to the longitudinal element.

X1.9 These test methods are not intended to allow direct comparison between the *in-vitro* results and clinical results. This is due to a number of factors including, but not limited to, the test fixture materials, lack of physiologic fluids, applied loads, clinical assemblage of constructs, over tightening screws into the UHMWPE blocks such that they buttress tightly against the plastic, and so forth. In order to better mimic clinical failures seen with some devices, it is suggested that the researcher investigate means of testing which would create the type and locations of failures seen clinically. Several possible modifications to the test methods and materials include inserting the screw such that its head does not tightly touch the UHMWPE block, counterboring the screw insertion holes 1 mm larger than the outer diameter of the screws for a depth equal to the screws' diameter, or the addition of soft washers with oversized holes. Any modification to the test methods and materials should maintain the specified block moment arm.

X1.10 These test methods are not intended to define performance levels of spinal implants as insufficient knowledge is available to predict the consequence of the use of particular spinal implant design and assemblies.

X2. ALTERNATIVE TEST METHODS

X2.1 The purpose of these test methods is to provide a means for comparison between spinal implant assemblies via a consistent testing method. The pinned block method in the body of these test methods constrains three degrees-of-freedom of superior relative to inferior vertebrae: lateral translation, lateral rotation about the (AP) *X* axis, and axial rotation about the *Z* axis. In certain testing applications, particularly for non-symmetrical constructs, the pinned block constraints may obscure some potential modes of movement and failure (**2**).

X2.2 To provide greater freedom to the implant assembly in certain loading conditions, two alternate methods are defined in this appendix: sphere joint superior-inferior blocks (**3, 4**), and spherical gimbal superior block with push rod (**2**). No assumptions are made here regarding the physiological relevancy of the three methods. It is up to the user to choose and report the method applied.

X2.3 The results of using the alternate methods may not be directly comparable to the results using the pinned block method due to changes in the vertebrectomy model mechanics (**4**). Although the moment arm between the force application and the screw entry is the same for all three methods, the change in testing mechanics, particularly constraints on the construct, may produce significant differences in test results for some modes of loading and construct configurations (**4**).

X2.4 *Sphere Joint Superior-Inferior Blocks*—Alternative sphere joint blocks for lumbar constructs, made of UltraHigh Molecular Weight Polyethylene (UHMWPE), are shown in Fig. 16. A 15.9 mm (5⁄8 in.) diameter spherical socket is shown to maintain the 12 mm longitudinal distance that was used for the pinned block in Fig. 13. Similarly, blocks for cervical constructs require a 12.7 mm (1⁄2 in.) diameter spherical socket to accommodate smaller size of the block (Fig. 7). Spherical blocks are limited to conducting compression-flexion tests.

X2.5 *Spherical Gimbal Superior Block with Push Rod*—A spherical gimbal superior block with push rod system similar to that used in Test Methods F2077 could be used as an alternative to perform unconstrained torsion as well as compression-flexion tests. This type of fixture arrangement for testing spinal implant constructs in a vertebrectomy model was proposed and illustrated by Carson (**2**)(Fig. 5).

REFERENCES

(1) Cunningham, B. W., Sefter, J. O., Shono, Y., and McAfee, P. C., "Static and Cyclical Biomechanical Analysis of Pedicle Screw Spinal Constructs," *Spine*, Vol 18, No. 12, pp. 1677–1688.

(2) Carson, W. L., "Relative 3 Dimensional Motions between End Vertebrae in a Bi-level construct, the Effect of Fixture Constraints on Test Results," *Spinal Implants: Are We Evaluating Them Appropriately, ASTM STP 1431*, M.N. Melkerson, S.L. Griffith, and J.S. Kirkpatrick, Eds., ASTM International, 2003.

(3) Cunningham, B. W., Sefter, J. C., Shono, Y., McAfee, P. C., "Static and Cyclical Biomechanical Analysis of Pedicle Screw Spinal Constructs," *Spine*, March 15, 2000, Vol 25, No. 6S, pp. 1S-12S.

(4) Dunbar, W.L., Cesarone, D., Serhan, H., "An Evaluation of the Influence of UHMWPE Test Block Design on the Mechanical Performance of Bilateral Lumbar Corpectomy Constructs," *Spinal Implants: Are We Evaluating Them Appropriately, ASTM STP 1431*, M.N. Melkerson, S.L. Griffith, and J.S. Kirkpatrick, Eds., ASTM International, 2003.

(5) Stanford et al. " Multiaxial Pedicle Screw Designs: Static and Dynamic Mechanical Testing," *Spine*, Vol 29, No. 4, 2004, pp. 367–375.

ASTM International takes no position respecting the validity of any patent rights asserted in connection with any item mentioned in this standard. Users of this standard are expressly advised that determination of the validity of any such patent rights, and the risk of infringement of such rights, are entirely their own responsibility.

This standard is subject to revision at any time by the responsible technical committee and must be reviewed every five years and if not revised, either reapproved or withdrawn. Your comments are invited either for revision of this standard or for additional standards and should be addressed to ASTM International Headquarters. Your comments will receive careful consideration at a meeting of the responsible technical committee, which you may attend. If you feel that your comments have not received a fair hearing you should make your views known to the ASTM Committee on Standards, at the address shown below.

This standard is copyrighted by ASTM International, 100 Barr Harbor Drive, PO Box C700, West Conshohocken, PA 19428-2959, United States. Individual reprints (single or multiple copies) of this standard may be obtained by contacting ASTM at the above address or at 610-832-9585 (phone), 610-832-9555 (fax), or service@astm.org (e-mail); or through the ASTM website (www.astm.org). Permission rights to photocopy the standard may also be secured from the ASTM website (www.astm.org/ COPYRIGHT/).
